# Oxygen Vacancy‐Mediated Bi─O Unsaturation Coordination in BiOCl for Efficient Photocatalytic Water Purification

**DOI:** 10.1002/advs.76301

**Published:** 2026-06-28

**Authors:** Shuaihao Ma, Jianglong Kong, Shidong Zhang, Wentao Li, Ling Yan, Deng Long, Xinglin Yu, Dawei Wang, Zheng Han, Sihan Ma, Lin Wang, Guang Ran

**Affiliations:** ^1^ College of Big Data and Information Engineering Guizhou University Guiyang China; ^2^ Department of Food Nutrition and Safety/National R&D Center Herbal Medicine Processing College of Engineering China Pharmaceutical University Nanjing China; ^3^ College of Medicine Guizhou University Guiyang China; ^4^ Key laboratory of Plant Resource Conservation and Germplasm Innovation in Mountainous Region (Ministry of Education) School of Pharmaceutical Sciences Guizhou University Guiyang China; ^5^ Qujing University of Medicine & Health Sciences Qujing China; ^6^ Department of Oncology Zhongshan Hospital of Xiamen University School of Medicine Xiamen University Xiamen China; ^7^ College of Energy Xiamen University Xiamen China

**Keywords:** adsorption, charge density, chemical engineering, degree of unsaturation, homogeneous distribution, oxygen, photocatalysis, portable water purification, radical

## Abstract

The stable modulation of the local charge distribution behavior induced by unsaturation sites within photocatalysts continues to pose a significant challenge in the quest to achieve notable enhancements in photocatalytic activity. Herein, we harnessed the hydrogen annealing reduction technique to deliberately introduce oxygen vacancies (OVs) into the BiOCl lattice for constructing the OVs‐Bi‐O structure, which decreases the valence state of Bi, and diminishes the Bi─O coordination, further establishing a charge asymmetric region within the material. This distinctive structural arrangement facilitates the sufficient migration of electrons to adjacent Bi sites that are closely linked to the OVs, significantly promoting the capture capability of electrons, leading to more adsorption and activation of water and oxygen as well as the conversion of reactive oxygen radicals. The engineered OVs‐BiOCl variant showcases potential photocatalytic prowess, boasting a satisfactory photocatalytic application than that of its unmodified BiOCl counterpart. Under low‐light conditions, this variant impressively achieves a ∼98.1% removal efficiency for RhB, while concurrently achieving an almost complete elimination of *E. coli*. This finding presents an insightful approach for manipulating unsaturation coordination active sites through the strategic introduction of controllable defects and elucidates the impact of unsaturation coordination on photocatalytic efficiency.

## Graphical abstract

The incorporation of oxygen vacancies (OVs) presents a viable strategy for fine‐tuning the photocatalytic prowess of BiOCl. A reliable hydrogen annealing reduction technique is employed to precisely design OVs, thereby optimizing the light absorption capacity, as well as the efficiency of carrier migration and separation in BiOCl. This system deepens the understanding of the photogenerated carrier migration mechanism, providing a theoretical basis for the construction of high‐performance photocatalysts.

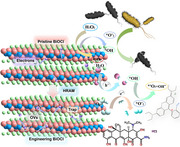

## 1 Introduction

In recent decades, with the rapid development of the manufacturing industry and healthcare industry, a large amount of harmful substances have entered water bodies, causing severe water pollution and damaging the ecological environment [1, 2, 3, 4, 5, 6, 7, 8, 9, 10]. Globally, roughly 80% of polluted water is discharged directly into water sources without undergoing any treatment. According to the WHO report, over half of the world's population will reside in regions lacking access to fresh drinking water [11]. Pollution is responsible for more than 5% of the GDP losses in developing countries [12]. Seriously, the persistent presence of water harmful microorganisms induced by the overuse of antibiotics poses a significant threat to the enduring sustainable development of society and the well‐being of life [13, 14, 15, 16]. Research reports reveal a staggering statistic: every year in Europe, over 25 000 people die from antibiotic‐resistant bacteria. In the United States, approximately 2.8 million people contract infections caused by drug‐resistant bacteria and over 35 000 of them die as a result [17]. If no measures are taken, by 2050, up to 10 million individuals will fall ill due to water harmful bacterial pollution [18]. Consequently, the imperative to develop superior water treatment technologies cannot be overstated.

Bismuth oxyhalides (BiOX, X = F, Cl, Br, I), as a new class of semiconductor materials, exhibit excellent photoelectric properties in photocatalytic degradation of organic pollutants and bacterial elimination [19, 20, 21, 22]. BiOX compounds consist of alternating [Bi_2_O_2_]^2+^ layers and double layers of halogen atoms, forming a layered crystal structure. Within such layered BiOX compounds, a unique internal electric field is generated [23, 24]. This built‐in field endows with anisotropic carrier transfer property, promotes the spatial separation of photogenerated electron‐hole pairs, thereby enabling BiOX to exhibit outstanding photocatalytic activity [25, 26]. Therefore, employing BiOX as a photocatalyst for the degradation of pollutants and the inactivation of harmful microorganisms in wastewater provides an effective approach to obtaining safe drinking water. However, the broad‐spectrum light utilization efficiency and poor interface polarization electric field of BiOX remain relatively low during the photocatalytic process, and the insufficient efficiency of photogenerated carrier separation severely restricts the generation of efficient photocatalytic performance. Oxygen vacancies (OVs), as deep electron traps [27], can be employed to effectively regulate the charge behavior on the catalysts and increase the light absorption capacity [28], and facilitate the efficient separation of photogenerated carriers, ultimately attaining outstanding photocatalytic performance [29]. While these defects are considered to be as a significant role in enhancing catalytic properties, an insufficient quantity is not conducive to the formation of the abundant active sites, and their excessive presence can destabilize the material by distorting local atomic arrangements, altering structural integrity, and modifying chemical reactivity, thereby reducing both stability and catalytic efficiency. Thus, the crux of boosting catalytic activity lies in effectively managing the intensity of defects to orchestrate the separation of photogenerated charges.

In this study, we have constructed BiOCl nanosheets with various adjustable defective sites by employing the hydrogen annealing reduction method (HARM). Compared with other conventional vacancy‐introduced approaches, such as high‐temperature calcination, ion doping, and chemical reduction, HARM possesses distinct superiorities: it enables accurate regulation of vacancy concentration by simply tuning annealing temperature, time, and hydrogen atmosphere ratio, avoids introducing impurity elements and complex post‐treatment procedures, and maintains the intrinsic crystal structure of BiOCl to the greatest extent. Moreover, HARM can efficiently induce rich OVs to optimize electronic band structure, extend light‐response range and accelerate photogenerated carrier separation, making it a green, low‐cost, and universal route for defect engineering of BiOCl photocatalysts. Experimental observations reveal that defective engineering, dominated by OVs, reduces the bandgap and enhances light‐harvesting efficiency in BiOCl‐based photocatalysts. Specifically, the introduction of controlled defects and their coordination with surrounding lattice atoms induces the local charge asymmetric distribution and the directional migration of electrons to facilitate efficient separation of photogenerated electron‐hole pairs, amplify surface local electric fields, and thereby significantly improve photocatalytic activity. Theoretical studies further confirm that the reasonable modulation of OVs lowers the adsorption energy of oxygen and water molecules, and improves the transfer pathway of electrons, which are critical for reactive oxygen species (ROS) generation. This reduction in energy barriers accelerates electron transfer, promoting the formation of abundant ROS intermediates. Compared to the pristine BiOCl, the HARM‐synthesized BiOCl with tunable defect parameters exhibits superior performance in both photodegradation and antibacterial applications. Photocatalytic degradation rates increased by three times despite at a lower light irradiation power (∼ 60 mW cm^−2^), and complete bacterial inactivation is achieved within 15 min of illumination. These findings highlight a scalable strategy for designing defect‐engineered photocatalysts with enhanced catalytic performance.

## 2 Results and Discussion

### 2.1 Morphological and Structural Characterization of BiOCl‐X

**FIGURE 1:**
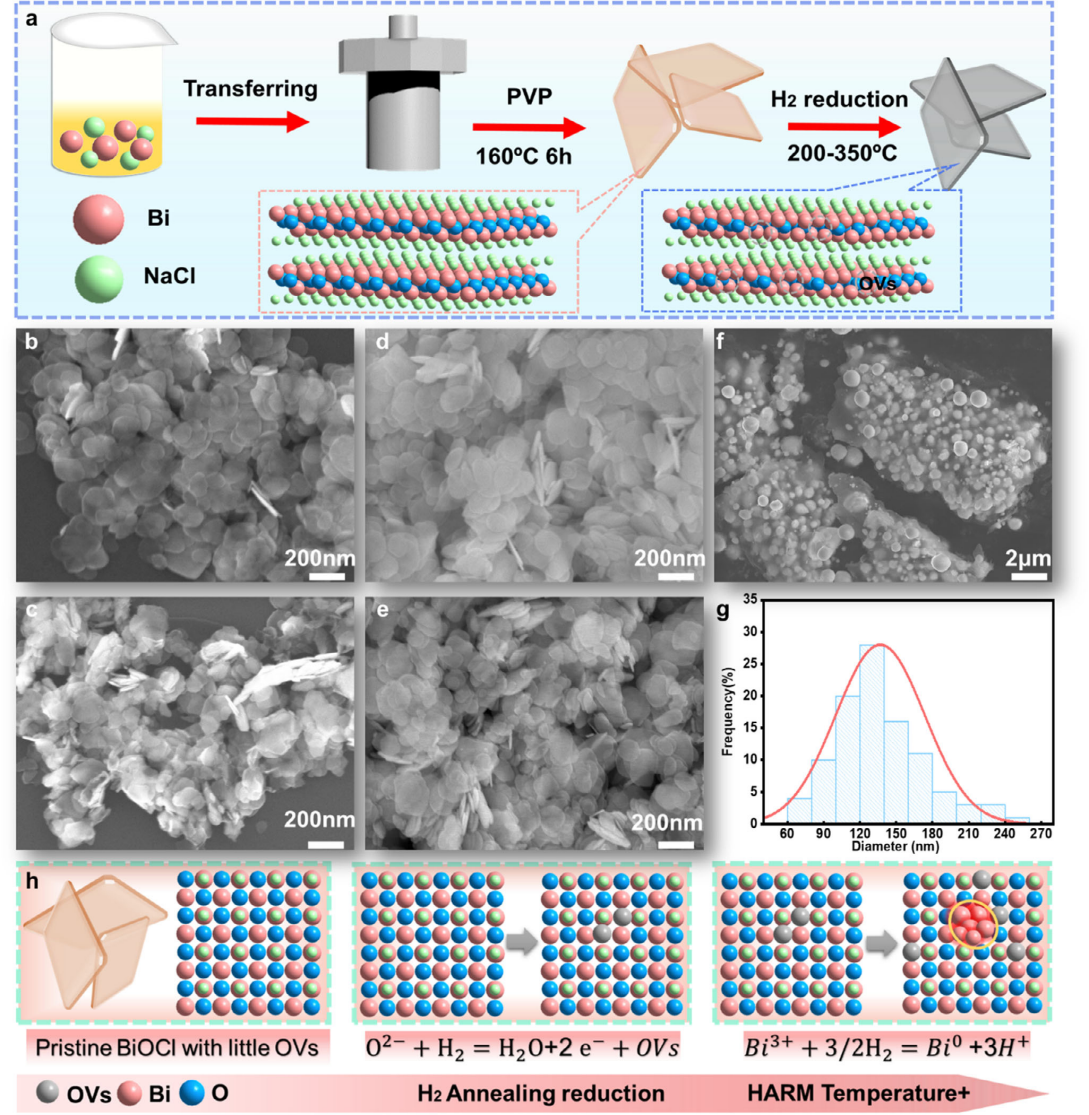
(a) Schematic illustration of the preparation of BiOCl‐X nanosheets. SEM images of (b) the pristine BiOCl, (c) BiOCl‐200, (d) BiOCl‐250, (e) BiOCl‐300, (f) BiOCl‐350, (g) Statistical analysis of the size of BiOCl nanosheets. (h) Schematic diagram for constructing the oxygen vacancies by HARM.

The pristine BiOCl was prepared by a hydrothermal strategy, and subsequently, HARM was employed to construct the BiOCl‐X (Figure 1a). During H_2_ reduction annealing at elevated temperatures, the color of pristine BiOCl underwent noticeable changes (Figure S1), suggesting that high‐temperature H_2_ treatment induces modifications in its local atomic structure and surface electronic properties. The observed color change is likely attributable to the formation of localized atomic vacancy defects, which alter the material's electronic structure and optical absorption properties [30]. The morphology of BiOCl‐X prepared by SEM characterization is shown in Figure 1b–f. EDS confirmed that the BiOCl prepared was mainly composed of elements of Bi, O, and Cl (Figure S2). The thickness of the lamellar BiOCl structure was precisely measured using Atomic Force Microscopy (AFM), as depicted in Figure S3. The BiOCl sample prepared exhibited a thickness of approximately 12 nm, closely aligning with the dimensions of the nanosheets observed in the SEM image. Both the as‐prepared BiOCl and the sample subjected to HARM at 300°C retained well‐defined lamellar structures, with BiOCl nanosheets exhibiting a narrow size distribution centered around 150 nm and high morphological uniformity (Figure 1g). Notably, when the annealing temperature was elevated to 350°C, excessive reduction occurred, leading to the formation of Bi nanospheres due to the complete reduction of BiOCl (Figure 1f). This phenomenon arises because oxygen atoms within the BiOCl lattice become thermally unstable at elevated temperatures, facilitating their reaction with hydrogen gas and subsequent defect formation. As the annealing temperature increases, the interaction between hydrogen molecules and BiOCl lattice atoms intensifies, promoting the stepwise reduction of Bi^3+^ to Bi^0^ via hydrogen (Figure 1h) [31]. This reduction process culminates in the nucleation and growth of metallic Bi nanospheres. Comprehensive analysis of X‐ray diffraction (XRD) patterns and X‐ray photoelectron spectroscopy (XPS) spectra provides conclusive evidence for the occurrence and mechanistic validity of this reduction process. As shown in Figure S4, the XRD spectrum reveals that the initially synthesized BiOCl structure exhibits a tetragonal phase. Notably, when employing HARM at temperatures beneath 300°C, the impact of varying temperatures on the BiOCl structure is negligible. However, an intriguing transformation occurs as the temperature climbs to 350°C, at which point the BiOCl structure morphs into that of the Bi material, providing further evidence of lattice atomic restructuring during the HARM process. The local structural images derived from XRD analysis reveal that, throughout the HARM process, as the temperature escalates, notable shifts in the characteristic peaks are discernible (Figure S5). These shifts are likely attributable to the ongoing generation of lattice vacancies. A meticulous examination of the O*1s* XPS spectrum reveals that, in comparison to the pristine BiOCl, the BiOCl‐X synthesized via the HARM process exhibits a higher concentration of defects. These defects are more likely to manifest themselves in the form of OVs. Furthermore, the proportion of the area where oxygen vacancies occur has increased, suggesting the emergence of more active sites for oxygen vacancies. This observation implies that the HARM technique is capable of promoting the generation of an increased number of defects across a range of temperature conditions (Figure S6). The pivotal observation here is that, when examining the distinct Bi*4f* peaks, the characteristic peaks of BiOCl‐X exhibit a shift toward higher binding energies in comparison to those of the original BiOCl (Figure S7). The corresponding Cl*2p* fine spectrum also shows similar characteristic peak shifts (Figure S8). This shift can be ascribed to the formation of oxygen vacancies and the corresponding electron redistribution on the catalyst surface. The generation of oxygen vacancies causes an increase in the local valence state of Bi species and a decrease in electron cloud density around Bi atoms, which strengthens the attraction of the nucleus to inner‐shell electrons and thus leads to the higher binding energy of Bi*4f*. This phenomenon further verifies the successful introduction of oxygen vacancies and the regulation of surface electronic structure, which is beneficial for optimizing the separation and transfer of photogenerated carriers [32]. The full‐spectrum scanning of XPS is presented in Figure S9. Lattice defects were vividly captured through HRTEM images, as depicted in Figure S10. The findings reveal that BiOCl adopts a tetragonal crystal structure, with the measured values of various crystal plane spacings aligning precisely with the standard spacings characteristic of BiOCl, corroborating the results derived from XRD analysis. Notably, BiOCl‐250 exhibits a substantially higher defect density compared to the original BiOCl. Furthermore, GPA analysis elucidates that BiOCl‐250 generates increased lattice stress, potentially stemming from an abundance of defects (Figure S11). These vacancies induce local lattice distortions, consequently leading to the generation of additional stress. The findings from electron spin resonance (ESR) spectroscopy unequivocally revealed that BiOCl‐250 harbored a greater number of OVs (*g =* 2.004) in comparison to the original BiOCl (Figure S12). The cumulative results outlined above have conclusively verified that the HARM technique is highly effective in generating OVs. Moreover, this method employs temperature‐regulated evaporation of gaseous atoms to facilitate the deposition of Bi atoms, which ultimately culminates in their transformation into Bi nanoparticles. These sequential processes align seamlessly with the morphological descriptions provided by SEM analysis.

**FIGURE 2:**
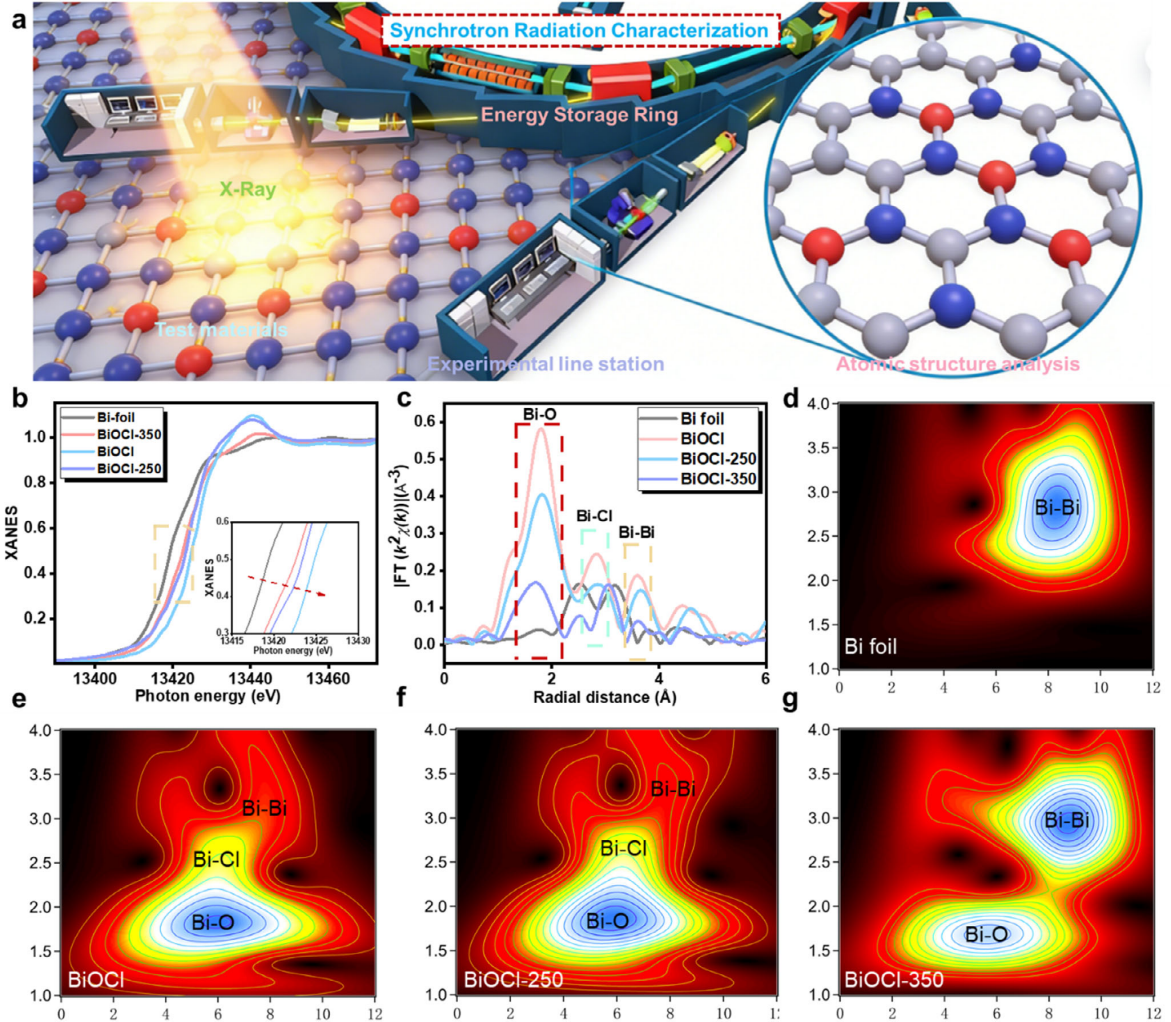
(a) Schematic illustration of XAS. (b) Bi L‐edge XANES, inset illustration comparing absorption intensity. (c) Fourier Transform EXAFS at Bi L‐edge. Corresponding wavelet transform of Bi L‐edge EXAFS of (d) Bi foil, (e) BiOCl, (f) BiOCl‐250, and (g) BiOCl‐350.

Synchrotron radiation‐based X‐ray absorption spectroscopy (XAS) can analyze the atomic coordination structure (Figure 2a) [33], and was employed to investigate the electronic structure and local coordination environment of Bi. As shown in Figure 2b, the observed absorption edge positions in E‐space reveal a systematic shift: BiOCl exhibits a higher edge energy compared to BiOCl‐250 and BiOCl‐350, both of which in turn show higher edge energies than metallic Bi foil. The X‐ray absorption near edge structure (XANES) spectra at the L‐edge of Bi demonstrate a direct correlation between the H_2_ processing temperature and the extent of Bi reduction, wherein elevated temperatures promote a more pronounced reduction of Bi species. The R‐space analysis of XAFS spectra provides critical insights into the local coordination environment of Bi (Figure 2c). For the as‐prepared BiOCl and BiOCl‐250 samples, the R‐space profiles exhibit distinct coordination peaks at approximately 1.8, 2.8, and 3.6 Å, corresponding to Bi–O first‐shell, Bi–Cl first‐shell, and Bi–Bi second‐shell interactions, respectively [34]. Notably, the BiOCl‐250 sample demonstrates a systematic attenuation in peak intensities across all coordination shells compared to BiOCl, indicative of reduced coordination numbers. This phenomenon is primarily attributed to the formation of OVs and chlorine vacancies during the reduction process, which disrupts the original coordination framework. In contrast, the BiOCl‐350 sample exhibits additional coordination features at 2.5 and 3.2 Å, similar to those observed in metallic Bi foil [35], which can be assigned to first‐shell Bi–Bi interactions. This observation suggests that under the 350°C reduction condition, BiOCl undergoes extensive reduction, leading to the nucleation and growth of elemental Bi precipitates. These results confirm that some vacancy defects induce the formation of O‐Bi‐OVs sites, accompanied by a decrease in the Bi valence state. The wavelet transform (WT) analysis of the Bi L‐edge EXAFS spectra (Figure 2d–g) reveals pronounced defect‐induced modifications in the local coordination environment of bismuth within the [Bi_2_O_2_]^2+^ layer. Specifically, the WT contour maps exhibit characteristic intensity modulations in the R‐space range of 2.5–3.5 Å, which are attributable to the emergence of high‐valent oxygen vacancy complexes in the layered structure. These defects trigger a charge compensation mechanism, wherein the oxidation state of adjacent Bi^3+^ is partially reduced, inducing the formation of Bi─Bi metallic bonds. This structural evolution is further corroborated by the concomitant enhancement of the Bi─Bi coordination peak intensity at 3.2 Å in the Fourier‐transformed EXAFS spectra. Based on the data from EXAFS (Figures S13–S16), a fitting analysis of R‐space was conducted to obtain the corresponding atomic structure information (Table S1). Furthermore, the EXAFS fitting curves of the research samples in the k‐space are shown in Figures S17–S21. The observed reduction in Bi─O coordination number and the concomitant elongation of Bi─Bi bond lengths can be primarily ascribed to two effects: (I) the stochastic variation in local OVs concentration, which directly modulates the coordination geometry around Bi sites, and (II) the OVs‐induced lattice strain field, which alters the electronic structure and subsequently reshapes the interatomic bonding interactions. The introduction of OVs induces notable alterations in the Raman spectrum [36]. As illustrated in Figure S22, Raman peaks at 144.4 and 200.4 cm^−1^ corresponding to the Bi─Cl stretching mode in BiOCl are discernible [32, 37]. The displacement of these peaks signifies a reduction in interlayer spacing, a phenomenon attributable to the formation of OVs within the Bi─O structure. The above structural studies demonstrate that the construction of OVs can be achieved through controllable HARM, thereby altering the local atomic coordination conditions.

### 2.2 Photochemical Analysis of BiOCl‐X

**FIGURE 3:**
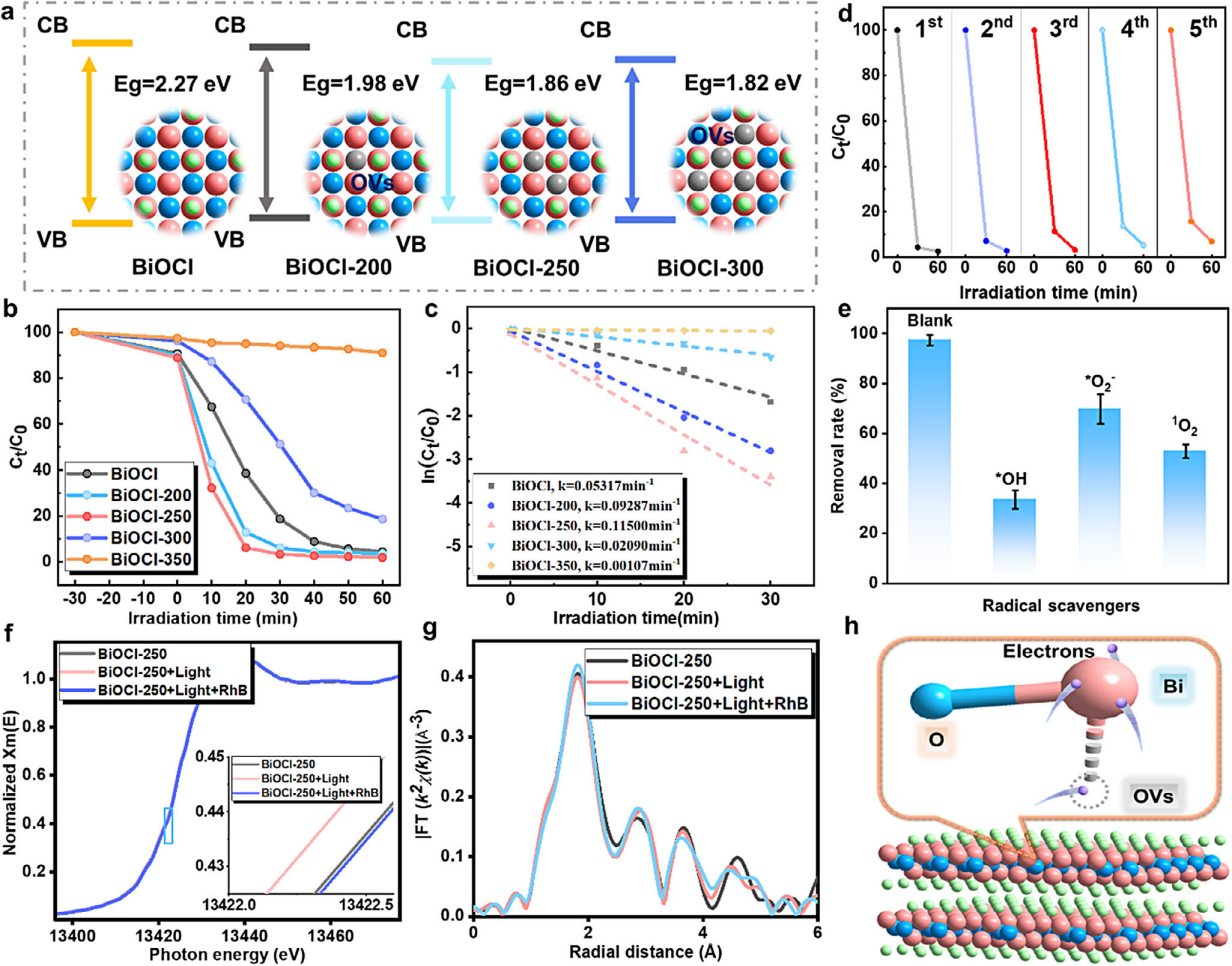
(a) Schematic illustration of the bandgap energy of the BiOCl and BiOCl‐X. (b) The photodegradation curves of RhB by different BiOCl‐X materials (Catalysts: 0.1g/L, RhB: 10mg/L). (c) The photodegradation kinetic curve of RhB. (d) The circulating degradation stability of RhB. (e) Free radical scavengers. L(+)‐Ascorbic acid, L‐Tryptophan, Tert‐butanol, and EDTA‐2Na were used as scavengers for •O_2_^−^, ^1^O_2_, •OH, and h^+^ species, respectively. (f) In situ XANES of BiOCl‐250. (g) In situ EXAFS of BiOCl‐250. (h) Schematic diagram illustrating the electronic migration and aggregation induced by unsaturated coordination.

The optical absorption properties of BiOCl‐X samples were investigated using UV–vis diffuse reflectance spectroscopy (DRS), with the results illustrated in Figure S23. The findings reveal that as the annealing temperature rises, the light absorption capacity of BiOCl extends across a broader spectral range. Calculations of the bandgap energies demonstrate that pristine BiOCl exhibits the widest bandgap at 2.27 eV, while the BiOCl samples annealed at 200°C to 350°C (BiOCl‐200 to BiOCl‐350) display progressively narrower bandgaps of 1.98, 1.86, 2.14, and 1.63 eV, respectively (Figure S24). Comprehensive theoretical computations further corroborate that the emergence of OVs plays a pivotal role in narrowing the bandgap, a finding that aligns harmoniously with the outcomes derived from experimental investigations (Figure S25). These results confirm that OVs exert a significant regulatory influence on the bandgap structure of BiOCl. Different concentrations of defects exhibit different bandgap widths (Figure 3a), and this result is theoretically consistent with some prominent experimental findings [38, 39, 40]. Ultraviolet photoelectron spectroscopy (UPS) was employed to determine the valence band maximum (VBM) potentials of the as‐prepared BiOCl and defective BiOCl samples. As shown in the UPS spectra (Figure S26), the VBM values, derived from the intersection of the baseline and the tangent of the secondary electron cutoff edge, were measured to be 1.69 eV for pristine BiOCl, and gradually decreased to 1.31, 0.83, and 0.39 eV for BiOCl‐200, BiOCl‐250, and BiOCl‐300, respectively. This downward shift of the VBM with increasing reduction temperature indicates that the introduction of oxygen vacancies effectively modulates the electronic structure of BiOCl, resulting in a continuous upshift of the valence band edge. BiOCl‐X exhibits enhanced light absorption capabilities, yet achieving optimal catalytic performance necessitates not only extended absorption but also efficient separation and migration of charge carriers. As depicted in Figure S27, the photocurrent response analysis reveals that BiOCl‐X demonstrates significantly improved photoelectric conversion efficiency compared to pristine BiOCl. Notably, BiOCl‐250 exhibits the most pronounced photocurrent enhancement, suggesting superior migration efficiency of photogenerated charge carriers within its structure. However, BiOCl‐300 demonstrates a diminished photocurrent response, likely due to an excessive concentration of defects that act as recombination centers by trapping a substantial number of electrons, thereby hindering the effective separation of photogenerated carriers. Meanwhile, BiOCl‐350, which has undergone significant reduction to form Bi nanoparticles, exhibits a narrower bandgap. This reduced bandgap not only shortens the carrier migration pathway but also increases the likelihood of electron–hole recombination, ultimately leading to a lower photocurrent density. As illustrated in Figure S28, the electrochemical impedance spectroscopy (EIS) analysis reveals a notable reduction in the impedance radius for BiOCl‐200 and BiOCl‐250 samples, signifying a decrease in surface charge transfer resistance across the modified BiOCl variants [41, 42, 43]. Notably, BiOCl‐250 exhibits the most efficient charge carrier mobility on its surface, demonstrating the fastest transfer rate within the tested medium. The increased impedance of BiOCl‐300 and BiOCl‐350 reduces the transmission efficiency of charge carriers, thereby affecting the photocatalytic activity. This phenomenon may be attributed to the annealing process, which causes the local vacancy defects on the surface to be passivated, reducing the lattice strain, but inhibiting the formation of ROS and the photocatalytic activity of BiOCl [44, 45]. As shown in Figure S29, BiOCl‐X demonstrates remarkable photothermal conversion capabilities, with its performance intensifying as the annealing temperature rises. This trend further corroborates the varying degrees of hydrogen reduction undergone by BiOCl at distinct temperatures. The presence of effective defects contributes to the narrowing of the bandgap, facilitating the conversion of surplus energy. When exposed to simulated sunlight, the photon energy of most incident light exceeds the bandgap of the BiOCl‐X samples, prompting the generation of electrons and holes above the bandgap within the semiconductor. As these charge carriers relax to the band edge, the excess energy is efficiently transformed into heat. The reduction in bandgap width enhances the relaxation process of electrons and holes, enabling a greater conversion of light energy into thermal energy [46, 47]. This efficient photothermal conversion mechanism can boost the photocatalytic activity of the materials.

### 2.3 Degradation and Disinfection

With RhB serving as the representative model pollutant, the catalytic performance of BiOCl was thoroughly assessed. RhB was selected due to its prevalent occurrence in wastewater and its inherent resistance to conventional treatment approaches. When compared to its untreated counterpart, BiOCl, the treated BiOCl‐250 demonstrates superior photocatalytic prowess. Remarkably, within a mere 30‐min timeframe, it achieves near‐total degradation of RhB (Figure 3b). Moreover, its effective degradation kinetic constant registers at 0.115 min^−1^, marking a 2.3‐fold enhancement over that of the unmodified BiOCl (Figure 3c). Subsequently, a series of degradation experiments was carried out using varying concentrations of RhB to investigate the correlation between the photocatalyst's degradation rate and the pollutant concentration. At an initial RhB concentration of 20 mg mL^−1^, approximately 92% of the dye was degraded within a span of 40 min (Figure S30). Notably, the degradation kinetic constant was fourfold that of the pristine BiOCl (Figure S31), suggesting that at low RhB concentrations, both BiOCl‐250 and BiOCl could effectively harness the ROS generated by light to achieve commendable RhB degradation. However, as the pollutant concentration increased, BiOCl‐250 demonstrated a superior catalytic activity. It was able to harness more light energy and exhibited an enhanced photogenerated carrier activity, leading to the production of an abundance of ROS. Consequently, it achieved superior pollutant degradation compared to the original BiOCl. Even when the RhB concentration was further elevated to 40 mg mL^−1^, BiOCl‐250 maintained a high degradation efficiency, reaching approximately 85% (Figure S32). The kinetic constant in this scenario remained nearly four times that of the unmodified BiOCl (Figure S33), underscoring its robust performance across a range of pollutant concentrations. Within the water supply system, BiOCl‐250 demonstrates effective pollutant removal capabilities (Figure S34), thereby underscoring its remarkable photocatalytic performance and versatility across diverse environmental conditions. The recycling of catalysts is of paramount importance for fostering economic environmental stewardship and advancing sustainable development. After undergoing five successive cycles, BiOCl‐250 continues to exhibit a remarkable pollutant removal efficiency exceeding 90% (Figure 3d). The morphology (Figure S35) and crystal structure (Figure S36) of the samples after the cyclic test showed almost no significant changes, confirming their excellent stability and long‐term reusability. To ascertain the primary free radicals instrumental in the degradation process, a series of free radical quenching experiments was meticulously conducted. As shown in Figure 3e, in the degradation pathway, hydroxyl radicals play a dominant role, while the function of superoxide anion is the weakest. Employing electronic spin resonance (ESR), we further delved into the various types of ROS. The signal intensity of *OH generated by BiOCl‐250 far surpassed that of the original BiOCl (Figure S37), whereas the signal for *O_2_^−^ remained largely unchanged (Figure S38). Additionally, there was a marginal enhancement in the signal for ^1^O_2_ compared to BiOCl (Figure S39). These findings suggest that BiOCl‐250 is capable of producing a greater quantity of ROS, with *OH playing a pivotal role, thereby leading to superior pollutant degradation performance. These results align harmoniously with the outcomes of our free radical quenching and degradation experiments. During the process of generating ROS, BiOCl‐250 exhibits higher levels of *OH and ^1^O_2_ compared to the original BiOCl. This disparity can be ascribed to the distinct behaviors of adsorbed H_2_O and O_2_ throughout the activation process. Specifically, O_2_ interacts with protons in H_2_O, utilizing electrons and yielding both ^1^O_2_ and H_2_O_2_. Following this, H_2_O_2_ reacts with *O_2_^−^ to produce additional *OH and ^1^O_2_. Consequently, the reaction system witnesses an elevation in the concentrations of *OH and ^1^O_2_, accompanied by a slight change in anion levels (Figure S40). In line with numerous prior investigations, the kinetics of free radical conversion can be succinctly outlined as follows [13, 21, 48]. By comparing with the recent publication, it was confirmed that BiOCl‐250 exhibited excellent catalytic performance (Figure S41).

$$O_{2}+e^{-}\to \cdot O_{2}^{-}$$

$$H_{2}O+h^{+}\to \cdot OH+H^{+}$$

$$O_{2}+2e^{-}+2H^{+}\to H_{2}O_{2}$$

$$\cdot O_{2}^{-}+H_{2}O_{2}\to \cdot OH+OH^{-}{+}^{1}O_{2}$$

$$\cdot O_{2}^{-}+\cdot OH\to OH^{-}{+}^{1}O_{2}$$

$$\cdot O_{2}^{-}{+}^{1}O_{2}+\cdot OH+\frac{RhB}{TCH}\to CO_{2}+H_{2}O$$

**FIGURE 4:**
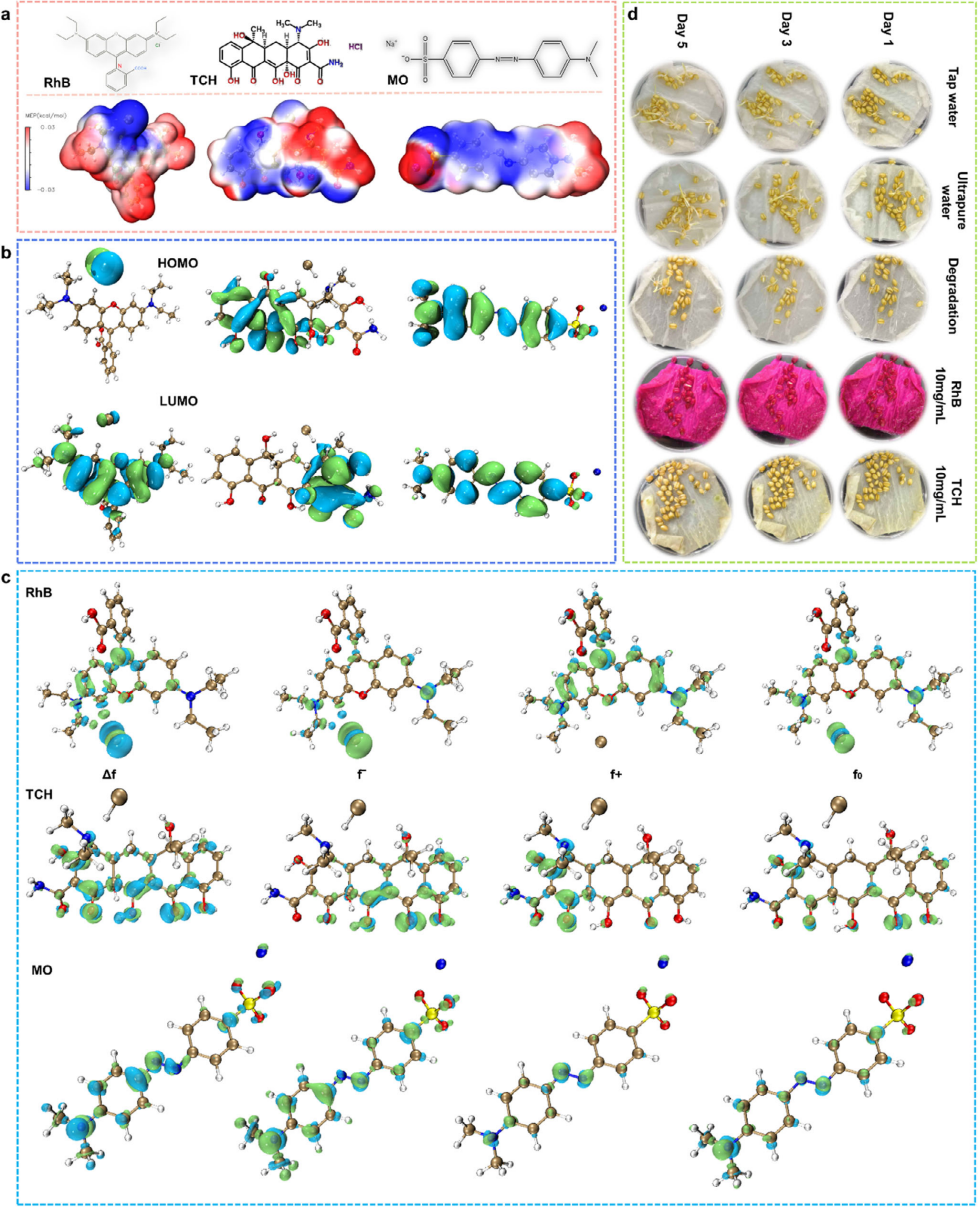
(a) Electrostatic potential (ESP) of RhB, TCH, and MO. (b) HOMO–LUMO distribution on various pollutants. (c) f^+^, f^−^, f^0^, and Δf distributions on various organic pollutants. (d) The growth status of wheat seeds in a mixed solution of deionized water, tap water, TCH, RhB, and photodegradation solution.

Furthermore, in situ X‐ray absorption spectroscopy was employed to delve into the electronic evolution behavior stemming from local structural alterations in BiOCl‐250 under illuminated conditions. As illustrated in Figure 3f, when viewed from the E‐space perspective, a notable shift in the absorption edge energy toward lower values was observed, signifying the aggregation of photogenerated electrons at the Bi sites. Upon the introduction of RhB and subsequent light exposure, the absorption edge position reverted to its original state with minimal deviation, implying the consumption of photogenerated carriers. From the R‐space perspective, the local coordination structure remained largely unaltered solely after light exposure (Figure 3g). However, following the addition of RhB and subsequent light exposure, the intensity of the Bi─O/N/C coordination peak within the first shell intensified, indicating the filling of OVs and an increase in the coordination number of Bi─O/N/C. These findings corroborate that the Bi─O unsaturated coordination, triggered by OVs, led to an accumulation of electrons around Bi atoms and underscored the role of defects in adsorbing active substances from the environmental medium (Figure 3h). Through meticulous fitting and comprehensive analysis of the R‐space and k‐space components of the absorption spectrum (Figures S42–S45), we successfully extracted coordination bond length and coordination number data that accurately reflect the true conditions represented by the dataset (Table S2). Under illuminated conditions, the coordination number exhibited minimal alteration, maintaining a relatively stable state. However, when subjected to reaction conditions, the coordination number of Bi─O underwent a discernible increase, rising from 3.0 to 3.2, which strongly suggests the adsorption of RhB molecules at the Bi site. The WT‐EXAFS integrates R‐space and k‐space data provides a nuanced, 3D perspective on the structural characteristics (Figure S46), and the Bi─O coordination analysis further reveals that the transfer and loss of electrons. Antibiotics, recognized as prevalent contaminants in water systems, have garnered significant research interest due to the pressing need for their efficient degradation. In this context, the degradation of TCH utilizing BiOCl‐250 was thoroughly examined. The findings revealed that around 60% of TCH underwent degradation within 1 h (Figure S47), accompanied by a degradation kinetic constant measured at 0.01337 min^−1^ (Figure S48). When benchmarked against the majority of photocatalysts documented in existing literature, it also demonstrates outstanding performance in the photodegradation of TCH (Figure S49). To ascertain the feasibility of the BiOCl‐250 system, a further assessment was conducted on its ability to degrade RhB under diverse conditions. Notably, when subjected to wastewater containing an array of ions, the catalytic system demonstrated a commendable degradation efficiency even after 60 min of treatment (Figure S50). Additionally, when employed for the photodegradation of MO, it showcases remarkable catalytic efficacy even under conditions characterized by low light intensity and minimal catalyst concentration (Figures S51 and S52 and Table S3). Meanwhile, by comparing and analyzing a previous type of BiOCl photocatalyst, the BiOCl‐OVs we designed still demonstrated satisfactory photocatalytic activity (Table S4). The corresponding degradation kinetic constants are presented in Table S5, demonstrating a competitive degradation trend. For various pollutant types, it becomes evident that the catalytic efficacy of BiOCl‐250 exhibits variability, potentially stemming from its distinct chemical structure and the spatial arrangement of surface electronic states. To gain a more profound insight into the degradation mechanisms underlying these three representative pollutants and to foster the advancement of high‐performance catalysts, we undertook theoretical investigations focusing on the surface electrostatic potential and the electronic behavior exhibited by the pollutants. The superior competitive degradation capability of RhB over TCH/MO was elucidated through an analysis of the ESP, as depicted in Figure 4a. The ESP serves as a fundamental physical parameter that characterizes the spatial distribution of potential energy within an electric field, directly reflecting the force exerted on each unit of positive charge across the given space. Regions with a high density of negative charge are particularly prone to attack by *OH and holes [49], whereas areas bearing a positive charge are more susceptible to assault from O_2_^−^. Consequently, RhB, which displays a greater density of negative charge, stands a higher chance of being preferentially degraded when *OH is present [50, 51, 52]. As shown in Figure 4b, the Highest Occupied Molecular Orbital (HOMO) of pollutant molecules represents the orbital with the highest energy that is occupied by electrons, making it inherently susceptible to electron loss. Consequently, it frequently serves as an electron donor in the process of photocatalysis. The photogenerated holes have the capability to directly extract electrons from the HOMO of the pollutant, thereby facilitating the oxidation of the pollutant. Simultaneously, the photogenerated electrons are channeled through the catalyst to the Lowest Unoccupied Molecular Orbital (LUMO) of the pollutant, enabling the occurrence of a reduction reaction [53, 54]. Furthermore, by using the Fukui function, the photocatalytic degradation active sites of different pollutants are analyzed (Figure 4c) [55], where f^−^, f^+^, and f° can quantitatively describe the possible reaction sites for electrophilic, nucleophilic, and radical reactions [56]. For RhB, the notably elevated f^−^ index, f^+^ value, and f^0^ index of 65(Cl) suggest that the diverse array of free radicals or reactive species generated can efficiently facilitate the degradation of RhB, exhibiting a non‐selective reactivity toward these active substances (Table S6). This characteristic is the underlying reason for RhB's heightened susceptibility to degradation. In the case of TCH, atoms 2(C), 15(C), 23(O), 33(O), and 55(O) demonstrate notably elevated f^−^ index values, signifying a pronounced sensitivity to electrophilic assault (Table S7). Conversely, atoms 41(O), 43(O), and 20(C) exhibit higher f^+^ values, rendering them susceptible to nucleophilic reagent attacks. Furthermore, atoms with higher f^0^ index values, including 20(C), 23(O), and 41(O), are particularly vulnerable to free radical‐induced degradation. Within the Fukui index framework for MO, there exists a comparatively limited number of active attack sites (Table S8). Atoms 23(N) and 13(C) stand out as being highly susceptible to electrophilic onslaughts. Meanwhile, atoms 12(N) and 36(Na) are inclined to undergo attacks from nucleophilic reagents. Furthermore, atoms 36(Na), 11(N), and 41(O) exhibit heightened vulnerability to free radical assaults. In essence, MO necessitates a more robust generation of ROS for effective degradation, thereby presenting a relatively challenging degradation profile. To evaluate the ecological toxicological effects of the products generated through photocatalytic conversion, we conducted a comparative analysis of plant toxicity. This analysis utilized several experimental groups, including ultrapure water, tap water, RhB (10 mg L^−1^), TCH (10 mg L^−1^), and pollutant solutions subjected to photodegradation (using our specifically designed BiOCl‐250 photocatalyst). Wheat seeds were cultivated hydroponically under strictly controlled conditions (10°C, in darkness) for a duration of five days, with samples collected and analyzed at scheduled intervals. The findings reveal that the experimental groups subjected to tap water and ultra‐pure water treatments exhibited a notably high germination rate. In stark contrast, the groups treated with RhB and TCH displayed minimal to no germination, underscoring their potent toxic effects on wheat. Notably, when compared to both water and RhB/TCH solutions, the germination rate witnessed a substantial increase following photocatalytic treatment (Figure 4d, Figure S53). This suggests a significant reduction in toxic substances post‐photocatalysis, thereby potentially diminishing the overall toxicity of the pollutants. The validation of the plant toxicity profile provides strong evidence for our BiOCl‐250 photocatalyst, supporting its potential for practical wastewater remediation applications.

**FIGURE 5:**
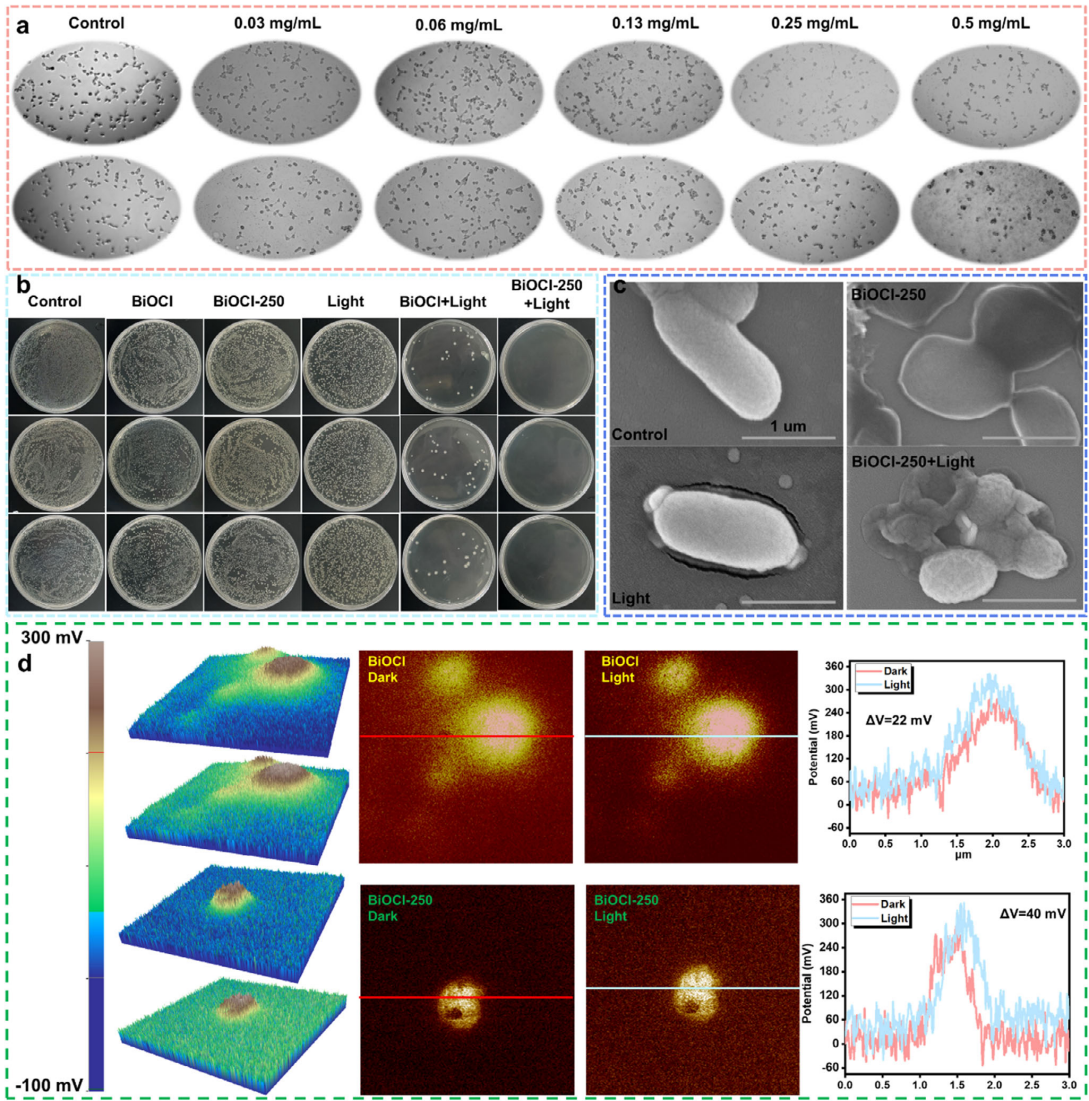
(a) Photographs of HEK293T incubated with BiOCl and BiOCl‐250. (b) Photographs of *E. coli* colonies with various treatment procedures. (c) SEM images of *E. coli*. (d) Surface potential images of BiOCl and BiOCl‐250.

In the course of photocatalytic water treatment, it is an unavoidable scenario that the photocatalyst will persist within the water body. To evaluate the potential accumulation toxicity of the photocatalyst in living organisms, the outcomes of the cytotoxicity test are presented in Figure 5a. With the escalation of BiOCl‐250 concentration, a discernible decline in cell density, coupled with morphological alterations is observed, indicating the onset of cell death. Specifically, at a high concentration of 0.5 mg mL^−1^, BiOCl‐250 manifests pronounced toxicity (Figure S54). Nevertheless, within a defined concentration range, the toxicity remains within acceptable limits. A comprehensive investigation was undertaken to explore the elimination of bacteria from contaminated water, with antibacterial experiments being conducted utilizing an optimally determined concentration of photocatalysts (Figure S55). By comparing, the concentration‐dependent bacterial inactivation property can be identified (Figure S56). As illustrated in Figure 5b, in stark contrast to the original BiOCl, BiOCl‐250 exhibited the capability to fully inactivate bacteria when exposed to light, thereby showcasing remarkable efficacy in the photodegradation‐mediated removal of *E. coli*. The antibacterial activity of the as‐prepared samples was further evaluated via live/dead fluorescence staining assays. In this test, viable bacterial cells emitted green fluorescence, whereas membrane‐damaged dead cells were labeled with red fluorescence. As presented in Figure S57, only green fluorescence was detected in the control groups under light irradiation alone or blank culture conditions, demonstrating that neither light illumination nor the sample background induced bacterial inactivation. By contrast, distinct and intense red fluorescence was observed when bacteria were incubated with the photocatalysts under light irradiation. Notably, the BiOCl‐250 group exhibited the strongest red fluorescence signal, implying the highest inactivation efficiency toward *E. coli*. This trend is highly consistent with the results of agar plate colony counting tests. Overall, the fluorescence characterization confirms that BiOCl‐250 possesses superior photocatalytic disinfection capability. The OD_600_ value reflects the survival status of bacteria under different treatment conditions, suggesting that BiOCl‐250 has excellent photocatalytic sterilization activity (Figure S58). The bacterial count reflects the excellent photocatalytic sterilization effect of BiOCl‐250 (Figure S59). The utilization of SEM to scrutinize the surface morphology of *E. coli*, we can gain preliminary insights into the antibacterial mechanism of photocatalysts (Figure 5c). Notably, the bacterial surface exhibits signs of damage, with localized structural collapses evident. This observation suggests a cascade of detrimental effects, including the inactivation of surface‐active substances on bacteria, the hindrance of electron conduction pathways, the disruption of cellular architecture, and the permeation of ROS through the compromised cell structure [57, 58, 59]. These ROS then interact with the genetic material within the bacteria, instigating DNA degradation and culminating in bacterial demise (Figure S60).

### 2.4 Photocatalytic Mechanism

**FIGURE 6:**
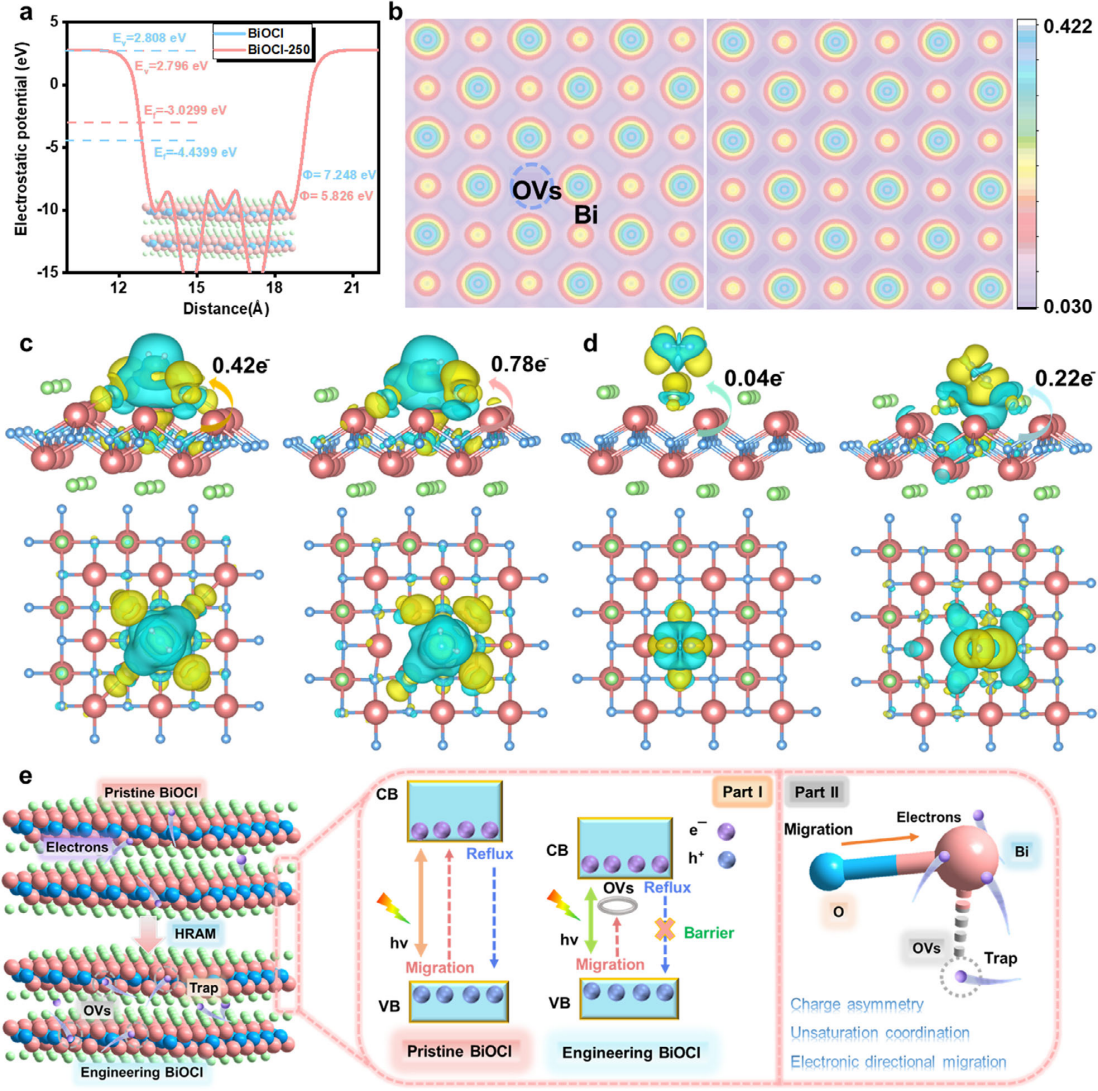
(a) Work functions of BiOCl and BiOCl‐250. (b) Surface average electric potential distribution. (c) Optimized charge density difference of H_2_O on BiOCl and BiOCl‐250. (d) Optimized charge density difference of O_2_ on BiOCl and BiOCl‐250 (yellow indicates the accumulation of electrons, while blue indicates the consumption of electrons). (e) Schematic diagram of enhanced photocatalytic activity for BiOCl‐250.

First, steady‐state photoluminescence (PL) analysis reveals that BiOCl‐250 demonstrates outstanding efficiency in separating photogenerated carriers (Figure S61). As illustrated in Figure S62, the findings on transient electron lifetime confirm that BiOCl‐250 possesses a prolonged fluorescence decay time, indicating a reduced likelihood of recombination among photogenerated electrons [60]. The surface electric potential distribution was meticulously examined utilizing a Kelvin probe force microscope (KPFM), as depicted in Figure 5d. Notably, the surface electric potential of the sample exhibited an increase both prior to and following illumination, suggesting the migration and accumulation of photogenerated electrons on the surface. Specifically, the surface potential difference observed for BiOCl‐250 was 40 mV, nearly double that of BiOCl. This finding further underscores its superior efficiency in separating photogenerated electrons, evidenced by the substantial migration and aggregation of electrons on the material's surface. Figure 6a illustrates the computed work functions for both BiOCl and BiOCl‐250. Notably, the work function of BiOCl‐250 is found to be lower than that of its pristine counterpart, BiOCl, suggesting the occurrence of charge transfer at the interface. This transfer subsequently leads to an elevation in the surface potential, which plays a pivotal role in bolstering the intrinsic electric field. Delving deeper through an examination of the surface average potential, it becomes evident that the incorporation of OVs augments the surface potential, thereby facilitating the migration of a greater number of electrons to the surface (Figure 6b). This migration, in turn, fortifies the separation efficacy of photogenerated carriers. These observations are in excellent agreement with the outcomes derived from KPFM analysis (Figure 5d). The introduction of OVs has led to the disorder of the local structure, which in turn has caused the asymmetry of the local charge distribution and also implies the emergence of charge redistribution behavior (Figure S63). Furthermore, the observed alterations in charge distribution also indicate that the emergence of OVs triggers a notable rise in electron density at the Bi sites, while concurrently causing a depletion of electrons surrounding the O atoms (Figure S64). This phenomenon unequivocally substantiates the exceptional proficiency of the system in separating photogenerated electrons. This result is in good agreement with the XAS results, verifying the validity of the experiments (Figure 3f,g). The density of electronic states reveals that the incorporation of OVs induces the formation of defect states, which in turn narrows the bandgap and broadens the spectrum of light absorption (Figure S65). The conduction band's position is predominantly shaped by the Bi *6p* orbitals, whereas the valence band's location is primarily influenced by the Cl *3p* and O *2p* orbitals. Consequently, upon illumination, electrons make a transition from the valence band to the conduction band, with the resulting photogenerated electrons predominantly accumulating at the Bi sites. Meanwhile, the photogenerated holes tend to gather at the O and Cl sites (Figure S66). Furthermore, the projected density of states (PDOS) for O_2_ and H_2_O molecules adsorbed onto the BiOCl surfaces was meticulously computed, with the results depicted in Figure S67. In addition, the electronic states of H_2_O exhibit no overlap with those of pristine BiOCl‐250. Conversely, the peaks corresponding to O_2_ align with those observed on the surfaces of BiOCl‐250. This observation strongly suggests that H_2_O molecules undergo physical adsorption on the BiOCl‐250 surface, whereas O_2_ engages in chemical adsorption when interacting with the surfaces of BiOCl‐250 [61]. These results further demonstrate that adsorbing H_2_O molecules on the BiOCl surface has a favorable activation effect to generate more *OH, and the results are consistent with those of ESR (Figures S37–S39).

An enhanced photocatalytic activity of the BiOCl‐250, which is further described by calculating the adsorption energy and the Bader charge transfer mechanism. As shown in Figures S68 and S69, when compared to the original BiOCl, BiOCl‐250 demonstrates a more pronounced negative adsorption energy for both H_2_O (−0.42 eV) and O_2_ (−0.23 eV), signifying that its interface possesses the highest adsorption capacity. This distinct adsorption preference governs the spatial arrangement of active molecules, leading to the accumulation of H_2_O and O_2_ on BiOCl‐250, thereby facilitating electron extraction and subsequent activation [62]. The findings depicted in Figure 6c reveal a notable accumulation of electron density at the Bi sites within BiOCl‐250. Concurrently, this pronounced electron buildup is predominantly localized around the adsorbed H_2_O molecules, particularly in the vicinity of the Bi─OVs bonds. Bader charge analysis further elucidates that the charge transfer from BiOCl and BiOCl‐250 to H_2_O amounts to 0.42e^−^ and 0.78e^−^, respectively. These observations imply that the incorporation of OVs facilitates a substantial injection of electrons into H_2_O, thereby fostering the formation of a greater abundance of ROS. As illustrated in Figure 6d, the electron transfer from BiOCl‐250 to O_2_ registers at 0.22e^−^, markedly surpassing the electron transfer from the pristine BiOCl (0.04e^−^). This indicates an expedited transformation process of the plentiful O_2_ molecules into active species. Collectively, these results unequivocally demonstrate that BiOCl‐250 is capable of generating a copious amount of ROS, thereby achieving commendable photocatalytic activity. Furthermore, a comprehensive technical and economic analysis (TEA) reveals that BiOCl‐250 stands out as a compelling substitute for traditional water pollution control methods or antibiotics, with a manufacturing cost of just 21.46$ g^−1^ (as illustrated in Figure S70 and Table S9). This underscores its remarkable economic feasibility. Collectively, these findings underscore that BiOCl‐250 not only excels in pollutant removal and boasts a favorable environmental impact but also offers cost‐efficiency, positioning it as a highly promising option for wastewater treatment endeavors.

## 3 Conclusion

In summary, we engineered a BiOCl‐250 catalyst featuring controllable OVs through the hydrogen reduction annealing technique, enabling dependable photocatalytic degradation and bacterial eradication. The strategic incorporation of these effective OVs fine‐tuned the local charge distribution asymmetry, established a localized Bi─O unsaturated coordination framework that fostered the concentrated accumulation of photogenerated electrons at Bi sites near the OVs, and amplified the adsorption capacity for water and oxygen molecules. This facilitated a more thorough interaction between charge carriers and the environmental reactive medium. Concurrently, the OVs trapped some electrons, synergistically enhancing the efficient separation of photogenerated carriers and, consequently, boosting photocatalytic activity. Our meticulously designed BiOCl‐250 demonstrated outstanding performance in photocatalytically degrading a variety of organic pollutants, achieving a photocatalytic activity threefold greater than that of the original BiOCl and effectively achieving near‐total bacterial inactivation. This endeavor not only introduces an efficient system for treating complex polluted wastewater but also offers inspired insights into the rational design of high‐performance catalysts.

## 4 Experimental Section

### 4.1 Materials

All chemicals currently used in our work are purchased from manufacturers (analytical grade) and can be used directly in experiments without further purification. Bismuth nitrate pentahydrate (Bi(NO_3_)_3_·5H_2_O, ≥99%) were obtained from Shanghai Aladdin Reagent Co., Ltd. Sodium chloride (NaCl, ≥99.5%) were purchased from Shanghai Titan Scientific Co., Ltd. Polyvinyl pyrrolidone (PVP) was purchased from Shanghai yuanye Bio‐Technology Co., Ltd.

### 4.2 BiOCl Nanosheets Fabricated by HARM

First, 2 mmol of Bi(NO_3_)_3_·5H_2_O was dissolved in 25 mL of deionized water and magnetically stirred for 30 min (denoted as solution A). Then, 2 mmol of NaCl and 0.1 g of PVP were dissolved in 25 mL of deionized water and magnetically stirred for 30 min (denoted as solution B). Solution B was slowly added dropwise to solution A. After magnetic stirring for 1 h to ensure a uniform mixture, the mixture was transferred into a reaction vessel and maintained at 160°C for 6 h. After reaction, the system was naturally cooled to room temperature, and the products were purified by centrifugation and washed three times with deionized water and ethanol. Finally, the obtained samples were dried at 50°C for 12 h to yield pure BiOCl nanosheets. Subsequently, the BiOCl nanosheets were annealed in a tube furnace. Purging the tube furnace with H_2_ at a flow rate of 50 sccm. After purging the residual air from the tube furnace, place the prepared BiOCl material in the tube furnace under a flowing H_2_ atmosphere. Roasting it at a heating rate of 10°C·min^−1^ to the specified temperature, hold the temperature for 60 min, and then allow it to cool naturally to ambient temperature to obtain BiOCl‐X (where X represents the holding temperature).

### 4.3 Characterization

The morphological characteristics of the material were thoroughly investigated through the use of scanning electron microscopy (SEM, HITACHI SU8010), transmission electron microscopy (TEM, TECNAI G20 F30 TWIN, Oxford), and atomic force microscopy (AFM). Its crystallographic structure was precisely determined through analyses performed with an X‐ray diffraction analyzer (XRD) and X‐ray photoelectron spectroscopy (XPS). The photoelectrochemical attributes were assessed using an electrochemical workstation, in conjunction with ultraviolet‐visible absorption spectroscopy (UV–vis abs). The carrier separation efficiency was tested using steady‐state‐transient fluorescence spectroscopy and Kelvin probe force microscope (KPFM). Additionally, the detection and examination of ROS were conducted utilizing an electron spin resonance spectrometer (ESR). The synchrotron radiation test makes use of the Shanghai Synchrotron Radiation Facility in China.

### 4.4 Photocatalytic Degradation

Preparing 100 mL of RhB solution in a beaker with a concentration of 10, 20, and 40 mg L^−1^, subsequently add BiOCl‐X with a dosage of 100 mg L^−1^. Allow adsorption in the dark for 1 h to ensure the adsorption‐desorption equilibrium is reached, then conduct the photocatalytic degradation experiment using a 75 W LED light source. Sampling every 10 min, centrifuge the samples, and determine the absorbance of the supernatant by ultraviolet‐visible absorption spectroscopy. The concentration of RhB can be obtained from a pre‐established calibration curve. The degradation efficiency of the reaction is calculated by the formula: Degradation efficiency = (C_0_−C_t_)/C_0_ × 100%, where C_0_ and C_t_ represent the initial concentration of the pollutant at the start of the photocatalytic reaction and the concentration at time t, respectively. To avoid interference from other light sources, the entire reaction is carried out under closed conditions. The degradation processes of tetracycline hydrochloride (TCH) and methyl orange (MO) should be carried out following the same procedure.

### 4.5 Photocatalytic Bactericidal Evaluation

Employing *E. coli* as the representative biological pollutant, the antibacterial efficacy of the membrane samples was systematically assessed through a range of techniques, including cloning culture, colony enumeration, fluorescence microscopy, and SEM characterization. The as‐prepared BiOCl samples were thoroughly blended with a bacterial suspension (OD_600_ = 1) and subsequently exposed to a 75 W LED illumination treatment. Following this treatment, the bacterial solution was incubated at 37°C for a duration of 2 h. Next, 100 µL of the incubated bacterial solution was extracted and diluted 1000‐fold using PBS. Thereafter, 100 µL of the diluted bacterial suspension was uniformly distributed across an LB agar plate. After an additional incubation period of 12 h at 37°C, the bacterial growth status within the LB medium was carefully observed and recorded. Subsequently, the treated bacteria were carefully placed onto a silicon wafer to facilitate detailed morphological observation using SEM, thereby enabling the determination of the bacterial inactivation pathway. Begin by taking 5 mL of the processed bacterial suspension and subjecting it to three successive washes with PBS. Subsequently, introduce the suspension into a fixation solution, such as paraformaldehyde or formalin, and allow it to fix for a duration of 20 min. Following fixation, thoroughly rinse the samples with PBS. Next, proceed with gradient dehydration of the fixed bacterial samples, employing a sequence of 25%, 50%, 75%, and absolute ethanol for the dehydration process. After completing the gradient dehydration, carefully transfer 3–5 µL of the bacterial liquid onto a silicon wafer and let it dry in an oven.

### 4.6 DFT Calculation Analysis

All calculations were performed using the Vienna Ab‐initio Simulation Package (VASP), which operates on first‐principles calculations grounded in density functional theory. To accurately describe electron exchange‐correlation, the generalized gradient approximation (GGA) was employed, incorporating the Perdew–Burke–Ernzerhof (PBE) exchange‐correlation potential. The electron wave functions were expanded using a plane wave basis set with a cutoff energy of 500 eV. Additionally, Becke–Johnson's DFT‐D3 method was applied to correct for van der Waals interactions between layers. For k‐point sampling in the first Brillouin zone, a Γ‐centered Monkhorst‐Pack scheme with 7 × 7 × 1 meshes was utilized. The model with a vacuum layer larger than 20 Å was adopted to calculate the electrostatic potential, and the dipole correction was applied. The convergence criterion for structural optimization is that the force on each atom in the model is less than 0.02 eV Å^−1^, and the total energy was converged to 10^−7^ eV.

### 4.7 Cytotoxicity Analysis

The cell line source was purchased from the National Infrastructure of Cell Line Resource, China. Hek293T cell viability was assessed using cell counting kit‐8 (CCK‐8; Dojindo Laboratories) according to the manufacturer's instructions. Briefly, cells were seeded in 96‐well plates (5 × 10^3^ cells/well), after various nanomaterial treatment (concentration: 0–0.5 mg/mL), cells were incubated with 10 µL CCK‐8 solution at 37°C for 2 h. Absorbance was measured at 450 nm using a microplate reader.

## Author Contributions

**Ling Yan**: methodology, investigation. **Deng Long**: formal analysis, validation. **Dawei Wang**: methodology, resources. **Shidong Zhang**: software, data curation, validation. **Guang Ran**: supervision, writing – review and editing, funding acquisition. **Lin Wang**: writing – review and editing, supervision. **Jianglong Kong**: conceptualization, methodology, validation, data curation, software. **Sihan Ma**: funding acquisition, supervision, writing – review and editing. **Wentao Li**: methodology, software. **Zheng Han**: visualization. **Shuaihao Ma**: conceptualization, methodology, software, data curation, investigation, writing – original draft. **Xin glin Yu**: formal analysis, investigation.

## AI Tool Disclosure

The atomic structure in Figure 2a was optimized via an open‐access Doubao AI tool, and the resulting graphic was combined with the schematic diagram of the synchrotron radiation facility. It is noteworthy that AI was only used for graphical refinement to enhance the interpretation of synchrotron technology, and no core research information was modified. The authors have completed a full review and scientific verification of all AI‐generated parts.

## Conflicts of Interest

The authors declare no conflicts of interest.

## Supporting information




**Supporting File**: advs76301‐sup‐0001‐SuppMat.docx.

## Data Availability

The data that support the findings of this study are available from the corresponding author upon reasonable request.

## References

[1] R. Yu , Y. Wang , W. Zhang , et al., “Induced Iron Vacancies Boosting FeOOH Loaded on Sustainable Fenton‐Like Collagen Fiber Membrane for Efficient Removal of Emerging Contaminants,” Applied Catalysis B: Environment and Energy 383 (2026): 126088, 10.1016/j.apcatb.2025.126088.

[2] Y. Zhou , W. Yang , L. Feng , J. Hong , M. Abbas , and S. Kawi , “Sunflower‐Disc‐Inspired Vertical Growth of 2D ZnIn_2_S_4_ on Ultra‐Thin TiO_2_: Constructing a 3D Porous Photocatalytic Glass Film for Ultra‐Efficient Organic Pollutant Degradation,” Applied Catalysis B: Environment and Energy 363 (2025): 124782, 10.1016/j.apcatb.2024.124782.

[3] Z. Zhou , S. Zhao , Z. Li , P. Wang , S. Zhan , and M. Wang , “Activating Oxygen via the 3‐Electron Pathway to Hydroxyl Radical by La‐O_4_ Single‐Atom on WO_3_ for Water Purification,” Angewandte Chemie International Edition 64 (2024): 202418122, 10.1002/anie.202418122. 39537570

[4] Y. Zhao , X. Fan , H. Zheng , E. Liu , J. Fan , and X. Wang , “Bi_2_WO_6_/AgInS_2_ S‐Scheme Heterojunction: Efficient Photodegradation of Organic Pollutant and Toxicity Evaluation,” Journal of Materials Science & Technology 170 (2024): 200–211, 10.1016/j.jmst.2023.06.022.

[5] R. Tai , S. Gao , Y. Tang , et al., “Defect Engineering of Bi_2_WO_6_ for Enhanced Photocatalytic Degradation of Antibiotic Pollutants,” Small 20 (2024): 2310785, 10.1002/smll.202310785. 38334181

[6] Y. Meng , Y.‐Q. Liu , C. Wang , et al., “Nanoconfinement Steers Nonradical Pathway Transition in Single Atom Fenton‐Like Catalysis for Improving Oxidant Utilization,” Nature Communications 15 (2024): 5134, 10.1038/s41467-024-49605-2. PMC11192908 38906879

[7] S. Ma , X. Yu , W. Li , J. Kong , D. Long , and X. Bai , “Bismuth‐Based Photocatalysts for Pollutant Degradation and Bacterial Disinfection in Sewage System: Classification, Modification and Mechanism,” Environmental Research 264 (2025): 120297, 10.1016/j.envres.2024.120297. 39515555

[8] S. Ma , X. Yu , Z. Han , et al., “BiO_2‐x_@Fe Nanospheres for Enhanced Degradation and Antibacterial Performances Using Synergistic Photocatalysis and Fenton Effect,” Ceramics International 51 (2025): 50875–50884, 10.1016/j.ceramint.2025.08.313.

[9] O. A. Sonmezoglu , A. Kamo , G. Karaaslan , B. Ercan , and S. Sonmezoglu , “In Situ Growth of S‐Scheme Zn_2_SnO_4_/Tb_2_O_3_ Heterostructures for Highly Efficient Visible‐Light‐Driven Photocatalytic Degradation and Bacterial Inactivation,” Applied Surface Science 717 (2026): 164688, 10.1016/j.apsusc.2025.164688.

[10] A. Kamo , O. A. Sonmezoglu , and S. Sonmezoglu , “Highly Efficient Photocatalyst Based on Zn_2‐x_BaxSnO_4_ Alloying Nanoparticles With Enhanced Photocatalytic Activity,” Inorganic Chemistry Communications 174 (2025): 114080, 10.1016/j.inoche.2025.114080.

[11] I. Jeon , E. C. Ryberg , P. J. J. Alvarez , and J. H. Kim , “Technology Assessment of Solar Disinfection for Drinking Water Treatment,” Nature Sustainability 5 (2022): 801–808, 10.1038/s41893-022-00915-7.

[12] A. I. Khdair , G. A. Aburumman , S. Gholipour , and M. Afrand , “Nanoparticles in Water Purification: Multifunctional Roles, Challenges, and Sustainable Applications,” Environmental Science: Nano 12 (2025): 3871–3895, 10.1039/d5en00268k.

[13] H. Yi , D. Ma , X. Huo , et al., “Facile Introduction of Coordinative Fe Into Oxygen‐Enriched Graphite Carbon Nitride for Efficient Photo‐Fenton Degradation of Tetracycline,” Journal of Colloid and Interface Science 660 (2024): 692–702, 10.1016/j.jcis.2024.01.131. 38271805

[14] X. Yu , Z. Wang , S. Kim , et al., “Boosted H_2_O_2_ Synthesis Performance of Supramolecular Porphyrin Homojunction Catalyst for Efficient Photo‐Self‐Fenton Elimination of Sulfonamide Antibiotics, Resistant Bacteria and Resistance Genes,” Applied Catalysis B: Environment and Energy 371 (2025): 125209, 10.1016/j.apcatb.2025.125209.

[15] J. Wang , J. Zhang , Y. Li , et al., “Silver Single Atoms and Nanoparticles on Floatable Monolithic Photocatalysts for Synergistic Solar Water Disinfection,” Nature Communications 16 (2025): 981, 10.1038/s41467-025-56339-2. PMC11761480 39856098

[16] O. A. Sonmezoglu , A. Kamo , and S. Sonmezoglu , “Phase‐Oriented Zinc Stannate Nanoparticles via Low‐Temperature Green Synthesis and Their Efficacy in Piezo/Flexo‐Phototronic Antibacterial Therapies,” Small 21 (2025): 06793, 10.1002/smll.202506793. 40970580

[17] S. Ma , Z. Zhao , S. Ma , D. Long , X. Yu , and W. Li , “Light‐Triggered Semiconductors for Bacterial Elimination,” Chemical Engineering Journal 506 (2025): 160243, 10.1016/j.cej.2025.160243.

[18] Z. Xiang , X. Wang , X. Jiang , et al., “Bismuth Oxyhalides (BiOX (X = Cl, Br, I)) Based Composites for Photocatalytic Antibacterial: Modification Strategies, Antibacterial Mechanisms and Prospects,” Journal of Environmental Chemical Engineering 13 (2025): 116428, 10.1016/j.jece.2025.116428.

[19] S. Ma , Y. Li , X. Luo , et al., “Dislocation‐Rich BiOI Nanosheets Designed by Nitrogen Ion Implantation for Improving NIR‐Triggered Bactericidal Activity,” Materials Today Energy 31 (2023): 101214, 10.1016/j.mtener.2022.101214.

[20] H. Zhao , X. Guan , F. Zhang , et al., “Rational Design of a Bismuth Oxyiodide (Bi/BiO_1‐x_I) Catalyst for Synergistic Photothermal and Photocatalytic Inactivation of Pathogenic Bacteria in Water,” Journal of Materials Science & Technology 100 (2022): 110–119, 10.1016/j.jmst.2021.05.056.

[21] Y. Zhong , S. Ma , D. Chen , et al., “Ultrathin BiOCl‐OV/CoAl‐LDH S‐Scheme Heterojunction for Efficient Photocatalytic Peroxymonosulfate Activation to Boost Co (IV) = O Generation,” Water Research 258 (2024): 121774, 10.1016/j.watres.2024.121774. 38772316

[22] B. Fu , Y. Pan , P. Zhao , et al., ““Edge In‐Situ Heterogeneous” BiOI Based on Defect Engineering and Non‐Noble Metal Deposition: Boosting Visible‐Light Photocatalytic Sterilization,” Chemical Engineering Journal 491 (2024): 152071, 10.1016/j.cej.2024.152071.

[23] X. Zhou , S. Zhang , W. Liu , J. Liu , and T. X. Liu , “In Situ Synthesis of Three‐Dimensional Core–Shell Structure Bi_2_WO_6_/BiOCl and Photocatalytic Degradation of Trinitrotoluene Wastewater,” Advanced Composites and Hybrid Materials 8 (2025): 102, 10.1007/s42114-024-01134-8.

[24] X. Wu , M. Shi , Y. Luo , et al., “Lanthanide Incorporation Orchestrates the Structure–Activity Relationship in Bismuth Oxychloride for Visible‐Light‐Driven Water Splitting,” Journal of the American Chemical Society 147 (2025): 43181–43192, 10.1021/jacs.5c17880. 41202152

[25] T. Peng , Y. Wang , C.‐L. Dong , et al., “BiOCl Atomic Layers With Electrons Enriched Active Sites Exposed for Efficient Photocatalytic CO_2_ Overall Splitting,” Nano‐Micro Letters 17 (2025): 223, 10.1007/s40820-025-01723-2. 40249405 PMC12008097

[26] P. Li , Y. Gao , A. G. Borthwick , et al., “Photocatalytic Nitrogen Reduction for Ammonia Synthesis Accelerated by Overcoming Photo‐Dember Effect,” Angewandte Chemie International Edition 137 (2025): 202503097, 10.1002/anie.202503097. 40200417

[27] A. Yamakata , K. Kato , T. Ogawa , et al., “Conduction Band and Defect Engineering for the Prominent Visible‐Light Responsive Photocatalysts,” Angewandte Chemie International Edition 64 (2025): 202419624, 10.1002/anie.202419624. 39714333

[28] A. Kamo , O. Ates Sonmezoglu , and S. Sonmezoglu , “Unraveling the Effects of Strain‐Induced Defect Engineering on the Visible‐Light‐Driven Photodynamic Performance of Zn_2_SnO_4_ Nanoparticles Modified by Larger Barium Cations,” ACS Applied Bio Materials 7 (2024): 8656–8670, 10.1021/acsabm.4c01447. 39556661

[29] R. Jiang , Y. Ji , M. Wang , Y. Chen , X. Wang , and G. Lu , “Degradation and Detoxification of Neonicotinoid Insecticides by a Porous Oxygen Vacancy‐Rich BiOCl Self‐Recovery System: Active Site Transfer Enhances Oxygen Vacancies Stability,” Water Research 282 (2025): 123651, 10.1016/j.watres.2025.123651. 40239377

[30] Y. Lu , Y. Huang , Y. Zhang , et al., “Oxygen Vacancy Engineering of Bi_2_O_3_/Bi_2_O_2_CO_3_ Heterojunctions: Implications of the Interfacial Charge Transfer, NO Adsorption and Removal,” Applied Catalysis B: Environmental 231 (2018): 357–367, 10.1016/j.apcatb.2018.01.008.

[31] Y. Chen , S. Chen , L. Zhang , et al., “Constructing 0D Bismuth‐Metal Nanosphere Networks on 1D/2D Bi_2_WO_6_ Heteromorphic Junctions for Efficient Photocatalytic Degradation of Acetaldehyde,” Advanced Materials 38 (2025): 13684, 10.1002/adma.202513684. 40944603

[32] Z. Wu , J. Shen , W. Li , et al., “Electron Self‐Sufficient Core‐Shell BiOCl@Fe‐BiOCl Nanosheets Boosting Fe(III)/Fe(II) Recycling and Synergetic Photocatalysis‐Fenton for Enhanced Degradation of Phenol,” Applied Catalysis B: Environmental 330 (2023): 122642, 10.1016/j.apcatb.2023.122642.

[33] Z. Xu , Q. Wang , L. Liu , et al., “Synergy of Local and Remote Electron Transfer Maintains the Electron Deficiency of Active Sites for Enhanced Photocatalytic Upcycling of Plastic Waste,” Journal of the American Chemical Society 147 (2025): 47605–47615, 10.1021/jacs.5c17129. 41396063

[34] Y. Zhang , Z. Xu , Q. Wang , et al., “Unveiling the Activity Origin of Ultrathin BiOCl Nanosheets for Photocatalytic CO_2_ Reduction,” Applied Catalysis B: Environmental 299 (2021): 120679.

[35] Q. Liang , J. Fan , X. Deng , et al., “Oxygen Vacancy‐Driven Asymmetrical Charge Distribution on Bi‐O‐Sn Sites in Sn‐Doped Bi_2_MoO_6_ for Efficient Photocatalytic CO_2_‐To‐CH_4_ Conversion,” Angewandte Chemie International Edition 138 (2025): 21874, 10.1002/anie.202521874. 41243684

[36] F. Mo , Z. Liu , and H. Li , “Synergistic Vacancy and Amorphization Engineering in BiOCl Heterostructures Enable Ultrafast Potassium‐Ion Storage,” Advanced Functional Materials 36 (2025): 13591, 10.1002/adfm.202513591.

[37] H. Sun , H. Lin , X. Jia , et al., “Dual Structure Cobalt Sites on Surface Hydroxyl and Oxygen Vacancy of BiOCl for Cooperative CO_2_ Reduction and Tetracycline Oxidation,” Applied Catalysis B: Environment and Energy 359 (2024): 124514, 10.1016/j.apcatb.2024.124514.

[38] J. Hou , D. Dai , R. Wei , et al., “Narrowing the Band Gap of BiOCl for the Hydroxyl Radical Generation of Photocatalysis Under Visible Light,” ACS Sustainable Chemistry & Engineering 7 (2019): 16569–16576, 10.1021/acssuschemeng.9b03885.

[39] G. Li , W. Yang , S. Gao , et al., “Creation of Rich Oxygen Vacancies in Bismuth Molybdate Nanosheets to Boost the Photocatalytic Nitrogen Fixation Performance Under Visible Light Illumination,” Chemical Engineering Journal 404 (2021): 127115, 10.1016/j.cej.2020.127115.

[40] C. Chen , T. Jiang , J. Hou , et al., “Oxygen Vacancies Induced Narrow Band Gap of BiOCl for Efficient Visible‐Light Catalytic Performance From Double Radicals,” Journal of Materials Science & Technology 114 (2022): 240–248, 10.1016/j.jmst.2021.12.006.

[41] X. Li , Q. Liu , F. Deng , et al., “Double‐Defect‐Induced Polarization Enhanced OV‐BiOBr/Cu_2−x_S High‐Low Junction for Boosted Photoelectrochemical Hydrogen Evolution,” Applied Catalysis B: Environmental 314 (2022): 121502, 10.1016/j.apcatb.2022.121502.

[42] C. Zhang , C. Xie , Y. Gao , et al., “Charge Separation by Creating Band Bending in Metal–Organic Frameworks for Improved Photocatalytic Hydrogen Evolution,” Angewandte Chemie International Edition 61 (2022): 202204108, 10.1002/anie.202204108. 35522460

[43] Q. Zhu , R. Hailili , Y. Xin , et al., “Efficient Full Spectrum Responsive Photocatalytic NO Conversion at Bi_2_Ti_2_O_7_: Co‐Effect of Plasmonic Bi and Oxygen Vacancies,” Applied Catalysis B: Environmental 319 (2022): 121888, 10.1016/j.apcatb.2022.121888.

[44] G. Kale , K. A. Ganure , A. Ghosh , S. G. Divakara , P. Dhanaraj , and S. V. Ganachari , “Annealing‐Induced Defect Modulation and Functional Switching in ZnO Thin Films: DFT and Molecular Docking Analysis,” Journal of Inorganic and Organometallic Polymers and Materials (2026): 1–3, 10.1007/s10904-025-04163-y.

[45] S. So¨nmezog˘lu , G. Çankaya , and N. Serin , “Influence of Annealing Temperature Onstructural, Morphological and Opticalproperties of Nanostructured TiO_2_ Thin Films,” Materials Technology 27 (2012): 251–256, 10.1179/1753555712Y.0000000008.

[46] Q. Wu , X. Liu , B. Li , et al., “Eco‐Friendly and Degradable Red Phosphorus Nanoparticles for Rapid Microbial Sterilization Under Visible Light,” Journal of Materials Science & Technology 67 (2021): 70–79, 10.1016/j.jmst.2020.04.084.

[47] J. Wang , Y. Li , L. Deng , et al., “High‐Performance Photothermal Conversion of Narrow‐Bandgap Ti_2_O_3_ Nanoparticles,” Advanced Materials 29 (2017): 1603730, 10.1002/adma.201603730. 27862379

[48] S. Gao , W. Liu , M. Wang , D. Fu , Z. Zhao , and X. Liu , “In Situ Self‐Grown Synthesis of c‐MOF@NiO Heterostructure Anchored to c‐MOF/rGA Particle Electrode: Promoting Sustained and Efficient Degradation of Phenol in Coking Wastewater,” Applied Catalysis B: Environment and Energy 365 (2025): 124911, 10.1016/j.apcatb.2024.124911.

[49] P. Zhao , J. Wang , D. Cao , et al., “Collagen‐Derived Nanoconfined Catalytic Membranes for Highly Efficient Water Remediation,” Advanced Materials 38 (2025): 19376, 10.1002/adma.202519376. 41388727

[50] J. Li , D. Wang , S. Zhao , et al., “Enhanced Peroxymonosulfate Activation by S‐Scheme AgI/Cu‐BiVO_4_ Heterojunction for Efficient Photocatalytic Organics Degradation and Microcystis Aeruginosa Inactivation: Performance, Interfacial Engineering and Mechanism Insight,” Applied Catalysis B: Environment and Energy 351 (2024): 124007, 10.1016/j.apcatb.2024.124007.

[51] S. Li , Y. Yang , J. Niu , et al., “Activation of PAA at the Fe–N_x_ Sites by Boron Nitride Quantum Dots Enhanced Charge Transfer Generates High‐Valent Metal‐Oxo Species for Antibiotics Degradation,” Environmental Science & Technology 58 (2024): 21871–21881, 10.1021/acs.est.4c08224. 39606938 PMC11709145

[52] C. Lin , G. Fan , J. Luo , C. Cai , X. Cao , and K.‐Q. Xu , “Synergistic Piezoelectric Effect and Oxygen Vacancies in MoS_2_/BiOIO_3_ Heterojunctions Boosting Photocatalytic Degradation of 17β‐Estradiol,” Chemical Engineering Journal 520 (2025): 166094, 10.1016/j.cej.2025.166094.

[53] W. Lian , P. Zhang , H. Che , B. Liu , and Y. Ao , “Efficiently Piezo‐Catalytic Generation of Reactive Oxygen Species on Phosphorus‐Doped BiOCl Enhancing Micropollutants Degradation,” Small 21 (2025): 2504949, 10.1002/smll.202504949. 40538260

[54] M. Fang , Z. Ning , H. Guo , et al., “Light‐Gated Thermal Domains in Nano‐Lanterns: Confined Heat Hotspots Sparks Electron Localization for Water Purification,” Advanced Science 13 (2025): 13730, 10.1002/advs.202513730. PMC12806286 41164989

[55] J. Wu , Y. Hu , Y. Huang , et al., “Sulfidation of Fenton‐Derived Iron Sludge to Activate Peroxymonosulfate for Enhanced Chloramphenicol Degradation: Mechanistic Insights and Life Cycle Assessment,” Water Research 290 (2026): 125120, 10.1016/j.watres.2025.125120. 41371072

[56] W. Zhu , Y. Cao , S. Li , and X. Li , “Degradation of Iohexol by Cu Ion‐Doped Bi_2_WO_6_ Activated Peroxydisulfate Under Synergistic Visible‐Light Photocatalysis/Piezoelectric Catalysis,” Chemical Engineering Journal 528 (2026): 172007, 10.1016/j.cej.2025.172007.

[57] D. Xia , Q. Chen , Y. Jiao , et al., “A Modified Flower Pollen‐Based Photothermocatalytic Process for Enhanced Solar Water Disinfection: Photoelectric Effect and Bactericidal Mechanisms,” Water Research 217 (2022): 118423, 10.1016/j.watres.2022.118423. 35417821

[58] Z. Zhang , J. Sun , S. Mo , et al., “Constructing a Highly Efficient CuS/Cu_9_S_5_ Heterojunction With Boosted Interfacial Charge Transfer for Near‐Infrared Photocatalytic Disinfection,” Chemical Engineering Journal 431 (2022): 134287, 10.1016/j.cej.2021.134287.

[59] X. Yang , Y. Ye , J. Sun , Z. Li , J. Ping , and X. Sun , “Recent Advances in g‐C_3_N_4_‐Based Photocatalysts for Pollutant Degradation and Bacterial Disinfection: Design Strategies, Mechanisms, and Applications,” Small 18 (2022): 2105089, 10.1002/smll.202105089. 34841656

[60] Y. Yang , Z. Du , H. Yang , et al., “Facet‐Engineered S‐Scheme Heterostructure With Enhanced Active Sites for Efficient Photocatalytic Degradation of Organic Contaminants,” Advanced Functional Materials 36 (2026): 25991, 10.1002/adfm.202525991.

[61] P. Chen , H. Liu , Y. Sun , et al., “Bi Metal Prevents the Deactivation of Oxygen Vacancies in Bi_2_O_2_CO_3_ for Stable and Efficient Photocatalytic NO Abatement,” Applied Catalysis B: Environmental 264 (2020): 118545, 10.1016/j.apcatb.2019.118545.

[62] T. Zhu , “An Efficient CuBi_2_O_4_/nZVI‐PEC/PMS System for Simultaneous Removal of OFL and Cr(VI): Synergistic Role of S‐Scheme/Ohmic Dual Junctions and Cu/Fe Double Active Centers,” Advanced Functional Materials 6 (2025): 22611, 10.1002/adfm.202522611.

