# Supplementary material for: Oxygen Vacancy‐Mediated Bi─O Unsaturation Coordination in BiOCl for Efficient Photocatalytic Water Purification
Source: Adv Sci (Weinh). 2026 Jun 28;13(52):e76301. doi: 10.1002/advs.76301 (PMC13336896; doi:10.1002/advs.76301)
Supplement: Supplementary file 1 [file ADVS-13-e76301-s001.docx]

**Oxygen Vacancy-Mediated Bi-O Unsaturation Coordination in BiOCl for Efficient Photocatalytic Water Purification**

Shuaihao Ma^a,#^, Jianglong Kong^b,#^, Shidong Zhang^a^, Wentao Li^a^, Ling Yan^c^, Deng Long^a^, Xinling Yu^a^, Dawei Wang^d^, Zheng Han^e^, Sihan Ma^a,*^, Lin Wang^f,*^, Guang Ran^g,*^

a. College of Big Data and Information Engineering, Guizhou University, Guiyang 550025, China

b. Department of Food Nutrition and Safety/National R&D Center Herbal Medicine

Processing, College of Engineering, China Pharmaceutical University, Nanjing 211198, China

c. College of Medicine, Guizhou University, Guiyang 550025, China

d. Key laboratory of Plant Resource Conservation and Germplasm Innovation in Mountainous Region (Ministry of Education), School of Pharmaceutical Sciences, Guizhou University, Guiyang, 550025, China

e. Qujing University of Medicine & Health Sciences, Qujing 655100, China

f. Department of Oncology, Zhongshan Hospital of Xiamen University, School of Medicine, Xiamen University, Xiamen 361004, China

g. College of Energy, Xiamen University, Xiamen 316002, China

Corresponding authors:

Sihan Ma: shma@gzu.edu.cn

Lin Wang: wanglin_linda82@163.com

Guang Ran: gran@xmu.edu.cn

**Supporting information**

**Fig S1. The photographs of the synthesized BiOCl-X.** The variations in color indicate that the surface of BiOCl has been reduced and local atomic defects have emerged.

**Fig S2. EDS mapping of the synthesized BiOCl.** The EDS analysis shows that the chemical composition of the surface and interior of the synthesis sample mainly consists of Bi, O, and Cl.

**Fig S3. Height of the synthesized BiOCl used by AFM.**

**Fig S4. XRD pattern diagram of the preparation of the BiOCl and BiOCl-X nanosheets.** The characteristic peaks of the prepared samples were well aligned with those of the standard PDF cards, indicating that the main crystal chemical structure of the compound was the one shown in the PDF. This further confirmed that the prepared compound was BiOCl. Under continuous reduction conditions, atomic defects were generated on the surface and inside of BiOCl, eventually leaving Bi atoms to recrystallize and form elemental Bi. This change can be observed from the XRD results.

**Fig S5. XRD local structure of the BiOCl and BiOCl-X.** The shift of the characteristic peaks may be attributed to the formation of defects. Hydrogen annealing at high temperatures does not result in hydrogen ion doping. The lattice atoms after reduction escape in gaseous form, leaving behind vacancies in the end.

**Fig S6. XPS O *1s* refine spectra of the BiOCl and BiOCl-X nanosheets.** The increase in the proportion of O defect area indicates that more oxygen vacancies have occurred.

**Fig S7. XPS Bi *4f* refine spectra of the BiOCl and BiOCl-X nanosheets.**

**Fig S8. XPS refine Cl *2p* spectra of the synthesized BiOCl and BiOCl-X.**

**Fig S9. XPS full spectra of the synthesized BiOCl and BiOCl-X.**

**Fig S10. HRTEM of (a) the pristine BiOCl. (b) IFFT image of BiOCl. (c) HRTEM image of the BiOCl-250. (d) IFFT image of BiOCl-250.**

**Note:** The lattice structure shown in Figure a indicates that it has a relatively complete crystal lattice atomic structure, with a small number of defects. As can be seen in Figure c, after hydrogen reduction, more lattice atomic defects appear, and the surface structure becomes blurred, indicating that the reduction has caused the destruction of the crystal lattice structure. The IFFT characterization also clearly shows that BiOCl-250 contains more defects.

**Fig S11. GPA images of the pristine BiOCl and BiOCl-250.**

**Note:** Phase analysis of geometric (GPA) is a microscopic strain analysis method, which is based on the principle that microscopic strains in samples will cause changes in lattice constants. By analyzing the microscopic strains present in the samples, we can further understand that the lattice distortion inside the samples may be caused by the introduction of vacancy defects. Effective defect formation will lead to changes in the local structure and generate stress, which is manifested as the generation of strain signals in GPA analysis.

**Fig S12. ESR detection of OVs.**

**Fig S13. Coordination bond fitting in the R-space of XAFS data for Bi foil.**

**Fig S14. Coordination bond fitting in the R-space of XAFS data for Bi in BiOCl-250.**

**Fig S15. Coordination bond fitting in the R-space of XAFS data for Bi in BiOCl.**

**Fig S16. Coordination bond fitting in the R-space of XAFS data for Bi in BiOCl-350.**

**Fig S17. FT-EXAFS fitting curves in k space of samples.**

**Fig S18. FT-EXAFS fitting curves in k space of Bi foil.**

**Fig S19. FT-EXAFS fitting curves in k space of BiOCl.**

**Fig S20. FT-EXAFS fitting curves in k space of BiOCl-250.**

**Fig S21. FT-EXAFS fitting curves in k space of BiOCl-350.**

**Fig S22. Raman spectra of BiOCl and BiOCl-250.** Raman peaks corresponding to the Bi-Cl stretching modes in BiOCl can be observed at 144.4, and 200.4 cm^−1^. In BiOCl, characteristic peaks of the Bi-Cl stretching mode were found. However, due to the introduction of defects, these two peaks shifted blue by approximately 4 cm^-1^.

**Fig S23. UV-vis DRS of the BiOCl-X.** The absorption edge redshift indicates an increase in the absorption range of visible light, corresponding to a decrease in the band gap width.

**Fig S24. Plot of αhυ versus energy (hυ) for the band gap energy of the preparation of the BiOCl and BiOCl-X nanosheets.**

**Fig S25. The calculated band structure and band gap.** When OVs are introduced, the band structure changes, and the reduction in band gap width leads to enhanced light absorption performance.

**Fig S26. UPS for different samples**. Indicating the valuable band maximum.

**Fig S27. Transient photocurrent spectra the BiOCl and BiOCl-X nanosheets.** Through the measurement of photocurrent, the degree of separation and migration of photogenerated carriers can be predicted. Under the same conditions, the higher the current intensity, the more electron migration, which indicates a good efficiency of photogenerated carrier migration. The fact that BiOCl-250 achieves the most significant photocurrent signal indicates that it has a better carrier mobility. In the subsequent evaluation, combined with the corresponding tests and analyses, a good catalytic effect can be obtained.

**Fig S28. EIS of the pristine BiOCl and the BiOCl-X.** The curvature radius shown by the Nyquist curve represents the characteristic of conductivity. A smaller radius indicates a lower impedance, and the formed electronic path becomes easier to achieve the conduction of electrons, confirming that BiOCl-250 has better conductivity to facilitate the good migration of charge carriers.

**Fig S29. Photothermal conversion and illustration of the BiOCl and BiOCl-X nanosheets.** Defect engineering can achieve enhanced photothermal conversion. BiOCl with a lower band gap can also achieve this enhanced photothermal conversion phenomenon. When photons with energy higher than the semiconductor band gap are radiated onto the semiconductor, it provides a strategy to generate electrons and holes above the band gap. Subsequently, the excited electrons or holes in the band gap relax to the edge of the band, and the excess relaxation can be converted into thermal energy. However, most of the light energy is absorbed by the semiconductor, and after electron-hole recombination near the band edge, it reappears as photons. Only a small portion of the light energy is converted into thermal energy through relaxation.

**Fig S30. Photodegradation curves of BiOCl-X (Catalysts: 0.1 g/L, RhB: 20 mg/L).**

**Note:** BiOCl-250 can completely degrade RhB within 40 minutes. The initial degradation efficiency of BiOCl was approximately 40%, indicating that BiOCl-250 achieves a better photocatalytic effect. This is likely due to the enhanced light absorption capacity and better migration efficiency of photogenerated carriers. These results are consistent with the photoelectrochemical performance test results in the experiment.

**Fig S31. Degradation dynamic curves of BiOCl-X (Catalysts: 0.1 g/L, RhB: 20 mg/L).**

**Fig S32. Photodegradation curves of BiOCl-X (Catalysts: 0.1 g/L, RhB: 40 mg/L).**

**Fig S33. Degradation dynamic curves of BiOCl-X (Catalysts: 0.1 g/L, RhB: 40 mg/L).**

**Fig S34. Degradation curve of RhB by BiOCl-250 in the water supply system.** It is suggested that for water pollution, BiOCl-250 can also achieve a good decontamination effect.

**Fig S35. SEM image of BiOCl-250 after five recycle degradation.** A good morphological structure is conducive to the exertion of photocatalytic activity. After five cycles of reuse, it still maintains a good structure, proving that BiOCl-250 can achieve long-term sustainable utilization.

**Fig S36. XRD pattern of BiOCl-250 after five recycle degradation.**

**Fig S37. ESR signals of DMPO-·OH with BiOCl and BiOCl-250.** ESR directly captures the signal of free radicals. The signal of BiOCl-250 is significantly higher than that of the original BiOCl, indicating that more hydroxyl free radicals can be generated under light conditions. This may be due to the improved structure of BiOCl-250, which leads to the formation of more photogenerated holes to facilitate the conversion of water, thereby further promoting degradation and antibacterial activity. The peak intensity of BiOCl-250 is 2.3 times that of the original BiOCl.

**Fig S38. ESR signal of *O_2_^-^ with BiOCl and BiOCl-250.** BiOCl-250 exhibited a relatively weak enhancement in the superoxide anion signal, indicating that the photogenerated electrons were effectively utilized during the formation of free radicals. However, all the electrons were utilized simultaneously and underwent conversion, forming other free radicals, thereby weakening the formation of superoxide anions.

**Fig S39. ESR signal of ^1^O_2_ with BiOCl and BiOCl-250.** The intuitive results show that the amount of singlet oxygen formed by BiOCl-250 is higher than that produced by the original BiOCl. By analyzing the ESR results of superoxide anions and hydroxyl radicals mentioned above, the superoxide anions produced by BiOCl-250 have not been significantly enhanced. This is attributed to the conversion of superoxide anions and the utilization of more electrons to generate singlet oxygen. The specific conversion pathway can be summarized as:

**Fig S40. Schematic illustration of ROS formation on BiOCl-250.**

**Note:** O_2_ reacts with protons in water, consuming electrons and generating ^1^O_2_ and H_2_O_2_. Subsequently, H_2_O_2_ reacts with *O_2_^-^ to produce *OH and ^1^O_2_. Therefore, the reaction system experiences an increase in the levels of *OH and ^1^O_2_, which in turn leads to a decrease in the concentration of anions.

**Fig S41. Comparison of k values with published literature of the catalytic materials for the RhB removal efficiencies (RhB: 20 mg/mL).** Through comparative analysis, BiOCl-250 can also achieve a good k value under the limited light power and catalyst concentration conditions. Compared with the currently reported BiOCl-10, it also demonstrates superiority. Because the implementation conditions of BiOCl-10 are 500W light and catalyst concentration of 0.4g/L, this means that the overall degradation conditions of BiOCl-250 are approximately 25 times lower than those of BiOCl-10. Therefore, under these conditions, BiOCl-250 can still achieve a degradation kinetic constant of 0.0675 min^-1^, demonstrating excellent performance.

**Fig S42. Coordination bond fitting in the R-space of In-situ XAFS data for Bi in BiOCl-250 under light irradiation.**

**Fig S43. Coordination bond fitting in the R-space of In-situ XAFS data for Bi in BiOCl-250 mixed with RhB under light irradiation.**

**Fig S44. In-situ FT-EXAFS fitting curves in k space of BiOCl-250 treated with light irradiation.**

**Fig S45. In-situ FT-EXAFS fitting curves in k space of BiOCl-250 mixed with RhB under illumination.**

**Fig S46. In-situ WT-EXAFS of BiOCl-250 treated with light irradiation.**

**Fig S47. Photodegradation curves of BiOCl-X (Catalysts: 0.1 g/L, TCH: 10 mg/L).** Compared with the degradation performance of RhB, TCH can also achieve 60% degradation within one hour. Although RhB is a triphenylmethane type dye, its chromophore structure is relatively simple and is more prone to ring-opening or decolorization reactions during photocatalysis. Clindamycin hydrochloride is a polycyclic aromatic compound with multiple conjugated rings and ionizable groups (such as amino and carboxyl groups), which has a stable structure and spatial steric hindrance effect, making it more difficult to be attacked by free radicals. Overall, it is possible to achieve complete degradation of TCH within the corresponding time frame by increasing the amount of light energy power (this work is 75W) and the usage of catalysts.

**Fig S48. Photodegradation dynamics of BiOCl-X (Catalysts: 0.1 g/L, TCH: 10 mg/L).**

**Fig S49. Analysis of TCH on various photocatalysts.** The relative superiority of the synthesized catalyst is demonstrated by comparing the data on TCH degradation in the literature. The concentration of TCH was all 10mg/mL. The photocatalytic effect varied under different light conditions and different catalyst concentrations. For the BiOCl-250 catalyst, although the degradation efficiency within a specific period was only about 60%, in this study, the catalyst concentration used was 0.1g/L and the light power was 75W. Compared with most catalysts and light powers, these conditions were all lower. Therefore, the photocatalytic effect of the BiOCl-250 catalyst was superior to that of most photocatalysts, proving a significant enhancement in its photocatalytic activity.

**Fig S50. RhB removal efficiency in the BiOCl-250 system under different anions at 60 min illumination.**

**Fig S51. Degradation curves of MO (Catalysts: 0.1g/L, MO: 10mg/L).**

**Fig S52. Degradation dynamic constants of various BiOCl (Catalysts: 0.1g/L, MO: 10mg/L).**

**Fig S53. Photographic of wheat growth under different treatments.** The growth of wheat seeds in untreated RhB and TCH solutions was significantly inhibited, and they were almost impossible to germinate. However, when the sewage treated by photocatalysis was used to cultivate wheat, the growth condition improved significantly, suggesting that photocatalysis can degrade harmful pollutants and reduce the impact of polluted water on plant growth.

**Fig S54. Cytotoxicity analysis of BiOCl and BiOCl-250 at different concentrations.** To achieve practical application and prevent ion leakage and residual enrichment of photocatalysts during the water treatment process, an acceptable cytotoxicity test is necessary. Using HEK293T cells as the receptor cells, the concentration-dependent cytotoxicity of photocatalysts was studied. The experimental results demonstrated that BiOCl exhibited good low toxicity at concentrations ranging from 0 to 0.5 mg/mL. For BiOCl-250, it showed significant cytotoxicity at a high concentration of 0.5 mg/mL. Therefore, when there is a low concentration of BiOCl-250 in the water body, good safety can be guaranteed.

**Fig S55. Photographs of *E. coli* colonies with different sample concentrations.** The antibacterial application of BiOCl-250 was carried out, with different concentration gradients set to ensure an appropriate photocatalyst concentration for achieving good antibacterial performance.

**Fig S56. OD_600_ value of *E. coli* colonies with different sample concentrations.**

**Fig S57. Live/dead fluorescence images of *E.coli* incubated with different sample concentrations.**

**Fig S58. OD_600_ value of *E. coli* colonies with different treatments.**

**Fig S59. Photographs of *E. coli* colonies with different sample concentrations.**

**Fig S60. Possible antibacterial mechanism of photocatalysts.** The death of bacteria is accomplished through the destruction of the cell membrane, the degradation of genetic material, and the inactivation of proteins, all of which result from reactive oxygen species with strong oxidizing capabilities.

**Fig S61. The steady-state photoluminescence (PL) of BiOCl and BiOCl-250.** The PL intensity of BiOCl-250 is lower than that of the original BiOCl, indicating that the recombination rate of photogenerated carriers in BiOCl-250 is significantly lower.

**Fig S62. time-resolved PL of BiOCl and BiOCl-250.** A longer carrier lifetime is conducive to the interaction with substances such as water and oxygen in the environmental medium, thereby inducing the production of more abundant ROS.

**Fig S63. The local charge density distribution of BiOCl.** The defect-free BIOCl exhibits a surface charge distribution with periodic symmetry. This means that when oxygen defects are introduced, the local periodic charge symmetry distribution will be disrupted, resulting in an asymmetric charge distribution.

**Fig S64. The differential charge density of BiOCl-250.** Electrons accumulate at the Bi site and are consumed at the O site, suggesting an effective electron transfer pathway.

**Fig S65. PDOS of BiOCl and BiOCl-250.**

**Fig S66. PDOS of Bi, O and Cl on BiOCl and BiOCl-250.**

**Fig S67. TDOS of H_2_O and O_2_ on BiOCl and BiOCl-250.** The physical state adsorption of H_2_O on the catalyst surface, thereby generating more hydroxyl radicals through a direct energy transfer process.

**Fig S68. Optimized model for H_2_O on BiOCl and BiOCl-250.**

**Fig S69. Optimized model for O_2_ on BiOCl and BiOCl-250.**

**Fig S70. Cost analysis of the prepared photocatalyst BiOCl-250.**

All the prices of chemical raw materials are sourced from this website: https://www.aladdin-e.com/zh_cn/b110815.html, the gas prices are purchased from the relevant enterprises: https://lem.gzu.edu.cn/2021/0825/c1234a156753/page.htm, The electricity rate is determined according to the local electricity policy (https://www.gzqz.gov.cn/zxfw/bmlqfw/sqfw/dfjf/).

**Table S1.** EXAFS fitting parameters at the Bi L-edge for various samples（*Ѕ*_0_^2^=0.780）

| Sample | Shell | *N^a^* | *R*(Å)*^b^* | *σ*^2^(Å^2^)*^c^* | Δ*E*_0_ (eV)*^d^* | *R* factor |
| --- | --- | --- | --- | --- | --- | --- |
| Bi foil | Bi-Bi | 4.0 | 3.58 | 0.0035 | 6.9 | 0.0047 |
|  | Bi-Bi | 6.0 | 3.84 |  |  |  |
| BiOCl | Bi-O | 4.1 | 2.27 | 0.0058 | -4.6 | 0.0047 |
|  | Bi-Cl | 4.0 | 3.05 | 0.0051 |  |  |
|  | Bi-Bi | 7.4 | 3.75 | 0.0025 |  |  |
| BiOCl-250 | Bi-O | 3.0 | 2.26 | 0.0047 | -4.5 | 0.0034 |
|  | Bi-Cl | 3.0 | 3.05 | 0.0033 |  |  |
|  | Bi-Bi | 6.4 | 3.81 | 0.0065 |  |  |
| BiOCl-350 | Bi-O | 1.2 | 2.21 | 0.0087 | 1.3 | 0.0078 |
|  | Bi-Cl | 2.5 | 3.47 | 0.0076 |  |  |
|  | Bi-Bi | 3.5 | 3.65 |  |  |  |

*^a^N*: coordination numbers; *^b^R*: bond distance; *^c^σ*^2^: Debye-Waller factors; *^d^* Δ*E*_0_: the inner potential correction. *R* factor: goodness of fit. *Ѕ*_0_^2^ was set to 0.780, according to the experimental EXAFS fit of Bi foil reference by fixing coordination numbers as the known crystallographic value.

**Table S2.** In-situ EXAFS fitting parameters at the Bi L-edge for various samples（*Ѕ*_0_^2^=0.780）

| Sample | Shell | *N^a^* | *R*(Å)*^b^* | *σ*^2^(Å^2^)*^c^* | Δ*E*_0_ (eV)*^d^* | *R* factor |
| --- | --- | --- | --- | --- | --- | --- |
| BiOCl-250 | Bi-O | 3.0 | 2.26 | 0.0047 | -4.5 | 0.0034 |
|  | Bi-Cl | 3.0 | 3.05 | 0.0033 |  |  |
|  | Bi-Bi | 6.4 | 3.81 | 0.0065 |  |  |
| BiOCl-250 +Light+RhB | Bi-O | 3.2 | 2.27 | 0.0070 | -4.6 | 0.0015 |
|  | Bi-Cl | 3.0 | 3.07 | 0.0026 |  |  |
|  | Bi-Bi | 6.3 | 3.94 | 0.0014 |  |  |
| BiOCl-250 Light | Bi-O | 3.0 | 2.26 | 0.0058 | -4.9 | 0.0057 |
|  | Bi-Cl | 3.1 | 3.09 | 0.0062 |  |  |
|  | Bi-Bi | 6.4 | 3.82 | 0.0033 |  |  |

*^a^N*: coordination numbers; *^b^R*: bond distance; *^c^σ*^2^: Debye-Waller factors; *^d^* Δ*E*_0_: the inner potential correction. *R* factor: goodness of fit. *Ѕ*_0_^2^ was set to 0.780, according to the experimental EXAFS fit of Bi foil reference by fixing coordination numbers as the known crystallographic value.

**Table S3.** Comparative analysis of photocatalytic degradation of MO

| Photoctalyst | Light time  (min) | Light power  (W) | MO concentration (mg/L) | Catalysts  concentration (g/L) | Degradation  rate | Ref |
| --- | --- | --- | --- | --- | --- | --- |
| CdSeBi_2_MoO_6_CSHs | 120 | 1000 | 10 | 0.3 | 98.5% | [15] |
| Bi_0.97_Bent_0.03_Fe_0.85_Co_0.15_O_3_ | 60 | -- | 5 | 0.5 | 92% | [16] |
| Ag-TP | 14 | 500 | 10 | 0.4 | 98.6% | [17] |
| rGO/Ag_3_PO_4_ | 90 | 300 | 10 | 0.5 | 97% | [18] |
| ZnCdS/MoO_3_ | 50 | 300 | 10 | 0.25 | 90.3% | [19] |
| MoS_2_/Co_3_O_4_ | 90 | 300 | 10 | 0.2 | 74.79% | [20] |
| BiOCl-250 | 60 | 75 | 10 | 0.1 | 30.6% | **This work** |

**Note:** Compared with the catalyst with the highest degradation efficiency shown in the table, the catalyst designed in this study still demonstrated relatively excellent MO degradation capability. With the catalyst dosage reduced by three times and the light power reduced by six times, 30% of MO was degraded within sixty minutes. Converted to the working conditions of the Ag-TP, BiOCl-250 could achieve 186% MO degradation, demonstrating the advanced nature of this catalyst.

**Table S4.** Comparative analysis of different BiOCl for degrading organic pollution.

| Photoctalyst | Light time  (min) | Light power  (W) | Pollution concentration (mg/L) | Catalysts  concentration (g/L) | Degradation  rate | Ref |
| --- | --- | --- | --- | --- | --- | --- |
| BiO_1−x_Cl-OH | 120 | 300 | 20 | 1 | 89.9% | [21] |
| BiOCl-OVs | 30 | -- | 5 | 0.5 | ~99% | [22] |
| AC-BiOCl | 20 | 300 | 10 | 0.5 | 97.5% | [23] |
| BiOCl (1 1 0) | 240 | 16 | 30 | 2 | 90% | [24] |
| BiOCl-20 | 60 | 500 | 10 | 0.5 | ~99% | [25] |
| BiOCl-250 | 60 | 75 | 10 | 0.1 | 30.6% | **This work** |

**Table S5.** The kinetics data of three organic pollutant degradation for BiOCl composites.

| **Catalysts** | **React constant of MO (min^-1^)** | | | **React constant of RhB (min^-1^)** | **React constant of TCH (min^-1^)** | |
| --- | --- | --- | --- | --- | --- | --- |
| BiOCl | | 0.00280 | 0.05317 | | | 0.00398 |
| BiOCl-200 | | 0.00402 | 0.09287 | | | 0.00832 |
| BiOCl-250 | | 0.00625 | 0.11500 | | | 0.01337 |
| BiOCl-300 | | 0.00184 | 0.02090 | | | 0.00281 |
| BiOCl-350 | | 0.00081 | 0.00107 | | | 0.00127 |

**Note:** Different pollution models have varying sensitivities to ROS, which is due to the different active sites targeted by ROS attacks. As a result, this leads to changes in the degradation reaction kinetics of different pollutants.

**Table S6.** Fukui function analysis of RhB

| **Atom** | **Q(N)** | **Q(N+1)** | **Q(N-1)** | **f^-^** | **f^+^** | **f^0^** | **CCD** |
| --- | --- | --- | --- | --- | --- | --- | --- |
| 1(C)  2(C)  3(C)  4(C)  5(C)  6(C)  7(C)  8(C)  9(C)  10(C)  11(H)  12(C)  13(C)  14(C)  15(H)  16(H)  17(H)  18(H)  19(H)  20(O)  21(C)  22(C)  23(C  24(C)  25(C)  26(H)  27(C)  28(H)  29(H)  30(H)  31(C)  32(O)  33(O)  34(H)  35(N  36(C)  37(H)  38(H)  39(C)  40(H)  41(H)  42(C)  43(H)  44(H)  45(H)  46(C)  47(H)  48(H)  49(H)  50(N)  51(C)  52(H)  53(H)  54(C)  55(H)  56(H)  57(C)  58(H)  59(H)  60(H)  61(C)  62(H)  63(H)  64(H)  65(Cl) | -0.0525  -0.0318  -0.0213  0.1035  -0.0676  0.0824  0.0458  0.1077  -0.0268  -0.0315  0.0444  -0.0606  0.0685  -0.0736  0.0427  0.0442  0.0339  0.0421  0.058  -0.0451  0.0197  -0.0131  -0.0315  -0.0266  -0.0219  0.0523  -0.0323  0.0497  0.0548  0.0529  0.2073  -0.263  -0.1713  0.1908  -0.0082  0.0098  0.0429  0.0353  0.0036  0.0344  0.0311  -0.0871  0.0357  0.0369  0.032  -0.0896  0.0335  0.0177  0.0443  -0.0356  0.0113  0.0384  0.0445  0.0057  0.0371  0.0391  -0.0792  0.0474  0.0346  0.0369  -0.0888  0.0353  0.0329  0.0368  -0.699 | -0.0931  -0.0713  -0.0399  0.0805  -0.0837  0.0465  -0.0341  0.0849  -0.0361  -0.0674  0.0262  -0.0932  0.0323  -0.0904  0.0218  0.0239  0.0267  0.0232  0.0449  -0.0768  0.0302  -0.0173  -0.0348  -0.0424  -0.0388  0.0481  -0.0542  0.0367  0.0403  0.0371  0.2036  -0.2597  -0.187  0.18  -0.0463  0.0023  0.0281  0.0262  -0.0031  0.0205  0.0266  -0.0955  0.0198  0.0283  0.0299  -0.0977  0.0326  0.0078  0.0312  -0.0634  0.0058  0.0289  0.0388  -0.0003  0.03  0.0275  -0.0854  0.0371  0.0272  0.0299  -0.0957  0.0278  0.0322  0.0229  -0.7408 | -0.0367  -0.0125  -0.0044  0.1102  -0.0545  0.0883  0.0631  0.1114  -0.0057  -0.0149  0.0564  -0.0335  0.0828  -0.0683  0.0553  0.058  0.0349  0.0559  0.0582  -0.0483  0.0102  -0.0126  -0.0321  -0.0171  -0.0128  0.0522  -0.0188  0.0575  0.0629  0.0629  0.2088  -0.2695  -0.1609  0.1968  0.0228  0.017  0.0533  0.046  0.0161  0.05  0.0339  -0.0782  0.0513  0.0424  0.0371  -0.0806  0.0365  0.0369  0.0509  -0.008  0.0145  0.0432  0.0463  0.0109  0.0439  0.048  -0.0746  0.0534  0.0412  0.0433  -0.0827  0.0401  0.0343  0.0484  -0.2607 | 0.0158  0.0193  0.0169  0.0067  0.013  0.006  0.0173  0.0037  0.0212  0.0166  0.012  0.0271  0.0143  0.0053  0.0125  0.0137  0.0009  0.0138  0.0002  -0.0032  -0.0095  0.0005  -0.0006  0.0095  0.0091  -0.0001  0.0135  0.0078  0.0081  0.01  0.0016  -0.0065  0.0104  0.0059  0.031  0.0072  0.0104  0.0107  0.0125  0.0157  0.0028  0.0089  0.0156  0.0054  0.0052  0.0089  0.003  0.0192  0.0066  0.0276  0.0033  0.0049  0.0018  0.0052  0.0069  0.0088  0.0045  0.006  0.0067  0.0064  0.0061  0.0048  0.0014  0.0116  0.4383 | 0.0406  0.0395  0.0186  0.0231  0.0162  0.0359  0.0799  0.0228  0.0093  0.0359  0.0183  0.0326  0.0362  0.0169  0.0209  0.0203  0.0073  0.0189  0.0131  0.0317  -0.0105  0.0041  0.0033  0.0158  0.0169  0.0042  0.0219  0.013  0.0145  0.0158  0.0037  -0.0033  0.0157  0.0108  0.0381  0.0076  0.0148  0.0091  0.0067  0.0138  0.0045  0.0085  0.016  0.0086  0.0021  0.0081  0.0009  0.0099  0.0131  0.0278  0.0055  0.0095  0.0056  0.0061  0.0071  0.0116  0.0063  0.0103  0.0073  0.0069  0.0069  0.0075  0.0007  0.0139  0.0418 | 0.0282  0.0294  0.0177  0.0149  0.0146  0.0209  0.0486  0.0132  0.0152  0.0262  0.0151  0.0298  0.0253  0.0111  0.0167  0.017  0.0041  0.0163  0.0067  0.0143  -0.01  0.0023  0.0014  0.0126  0.013  0.002  0.0177  0.0104  0.0113  0.0129  0.0026  -0.0049  0.0131  0.0084  0.0345  0.0074  0.0126  0.0099  0.0096  0.0148  0.0036  0.0087  0.0158  0.007  0.0036  0.0085  0.002  0.0145  0.0099  0.0277  0.0044  0.0072  0.0037  0.0056  0.007  0.0102  0.0054  0.0082  0.007  0.0067  0.0065  0.0062  0.001  0.0128  0.2401 | 0.0248  0.0202  0.0017  0.0164  0.0031  0.0299  0.0625  0.0191  -0.0119  0.0193  0.0063  0.0055  0.0219  0.0116  0.0084  0.0065  0.0063  0.0051  0.0129  0.0349  -0.0011  0.0036  0.0039  0.0063  0.0079  0.0043  0.0084  0.0052  0.0064  0.0058  0.0021  0.0032  0.0052  0.0049  0.0071  0.0004  0.0044  -0.0016  -0.0058  -0.0018  0.0017  -0.0004  0.0004  0.0032  -0.0031  -0.0009  -0.0021  -0.0093  0.0065  0.0002  0.0022  0.0046  0.0038  0.0009  0.0002  0.0028  0.0017  0.0044  0.0007  0.0005  0.0007  0.0027  -0.0007  0.0023  -0.3965 |

**Table S7.** Fukui function analysis of TCH

| **Atom** | **Q(N)** | **Q(N+1)** | **Q(N-1)** | **f^-^** | **f^+^** | **f^0^** | **CCD** |
| --- | --- | --- | --- | --- | --- | --- | --- |
| 1(C)  2(C)  3(C)  4(C)  5(C)  6(C)  7(C)  8(C)  9(C)  10(C)  11(C)  12(H)  13(C)  14(C)  15(C)  16(H)  17(H)  18(H)  19(C)  20(C)  21(C)  22(C)  23(O)  24(O)  25(H)  26(C)  27(H)  28(H)  29(H)  30(H)  31(H)  32(H)  33(O)  34(H)  35(O)  36(H)  37(H)  38(N)  39(O)  40(H)  41(O)  42(C)  43(O)  44(N)  45(H)  46(H)  47(C)  48(H)  49(H)  50(H)  51(C)  52(H)  53(H)  54(H)  55(O)  56(H)  57(H)  58(Cl) | -0.0349  -0.0604  0.0032  -0.0359  0.0875  -0.0759  0.0882  0.1305  -0.0504  -0.0276  -0.0601  0.0267  -0.0169  0.0702  0.0745  0.0463  0.039  0.0386  0.0316  0.1124  -0.072  0.1454  -0.2518  -0.2178  0.1518  -0.0942  0.0228  0.0356  0.0356  0.0314  0.0184  0.0435  -0.1839  0.1326  -0.2121  0.1692  0.048  -0.0377  -0.1584  0.1427  -0.2084  0.1766  -0.2565  -0.1398  0.1378  0.1313  -0.0372  0.045  0.0243  0.0406  -0.0375  0.0415  0.0454  0.025  -0.1726  0.1802  0.0843  -0.2156 | -0.0594  -0.0708  -0.0007  -0.0357  0.0767  -0.0919  0.0869  0.1065  -0.0704  -0.0304  -0.0642  0.0172  -0.0199  0.0622  0.072  0.0314  0.0306  0.0256  0.0224  0.0127  -0.1077  0.0747  -0.2912  -0.2266  0.1479  -0.0974  0.0211  0.0343  0.0245  0.0226  0.0149  0.0327  -0.1994  0.1245  -0.2373  0.1555  0.0301  -0.042  -0.2264  0.1226  -0.2972  0.1502  -0.31  -0.1596  0.113  0.1176  -0.0428  0.036  0.0208  0.0244  -0.0412  0.0404  0.0265  0.0234  -0.1805  0.1701  0.0832  -0.2525 | 0.0137  -0.0028  0.02  0.0021  0.1291  -0.0401  0.0926  0.1568  -0.0009  -0.0196  -0.0567  0.0385  -0.0154  0.0751  0.1268  0.0757  0.0659  0.0653  0.0338  0.1195  -0.067  0.1502  -0.1513  -0.1999  0.1649  -0.0884  0.0363  0.0366  0.0474  0.0491  0.0235  0.0496  -0.1328  0.1497  -0.1929  0.1759  0.055  -0.0389  -0.1421  0.1494  -0.1886  0.1796  -0.2451  -0.1318  0.1476  0.136  -0.0328  0.0501  0.0306  0.0475  -0.0362  0.0342  0.0521  0.0309  -0.1168  0.2082  0.0823  -0.2011 | 0.0486  0.0576  0.0168  0.038  0.0417  0.0358  0.0044  0.0263  0.0495  0.008  0.0034  0.0118  0.0015  0.0049  0.0523  0.0294  0.0269  0.0267  0.0022  0.0071  0.005  0.0048  0.1005  0.0179  0.0131  0.0058  0.0135  0.001  0.0117  0.0176  0.005  0.0061  0.0511  0.0171  0.0192  0.0066  0.007  -0.0012  0.0163  0.0067  0.0198  0.003  0.0114  0.008  0.0099  0.0047  0.0044  0.0051  0.0063  0.0069  0.0013  -0.0073  0.0067  0.0059  0.0558  0.0279  -0.0021  0.0144 | 0.0245  0.0104  0.0039  -0.0002  0.0107  0.016  0.0013  0.0241  0.02  0.0029  0.0041  0.0095  0.003  0.0081  0.0025  0.0148  0.0084  0.013  0.0091  0.0997  0.0356  0.0707  0.0394  0.0088  0.0039  0.0032  0.0017  0.0013  0.0111  0.0089  0.0036  0.0108  0.0154  0.0081  0.0252  0.0137  0.018  0.0043  0.068  0.0201  0.0889  0.0264  0.0535  0.0198  0.0247  0.0136  0.0056  0.009  0.0035  0.0161  0.0037  0.0011  0.019  0.0016  0.0079  0.0101  0.0011  0.0369 | 0.0366  0.034  0.0104  0.0189  0.0262  0.0259  0.0028  0.0252  0.0348  0.0054  0.0037  0.0107  0.0023  0.0065  0.0274  0.0221  0.0176  0.0198  0.0057  0.0534  0.0203  0.0378  0.0699  0.0133  0.0085  0.0045  0.0076  0.0011  0.0114  0.0132  0.0043  0.0084  0.0333  0.0126  0.0222  0.0102  0.0125  0.0015  0.0421  0.0134  0.0543  0.0147  0.0325  0.0139  0.0173  0.0092  0.005  0.0071  0.0049  0.0115  0.0025  -0.0031  0.0128  0.0037  0.0319  0.019  -0.0005  0.0257 | -0.0242  -0.0471  -0.0128  -0.0382  -0.031  -0.0199  -0.0031  -0.0022  -0.0294  -0.0051  0.0007  -0.0023  0.0016  0.0031  -0.0498  -0.0145  -0.0184  -0.0137  0.0069  0.0925  0.0306  0.0658  -0.0611  -0.0091  -0.0092  -0.0026  -0.0118  0.0003  -0.0006  -0.0088  -0.0015  0.0047  -0.0357  -0.009  0.0061  0.007  0.011  0.0056  0.0517  0.0133  0.0691  0.0234  0.0421  0.0118  0.0148  0.009  0.0012  0.0039  -0.0028  0.0092  0.0024  0.0084  0.0122  -0.0043  -0.0479  -0.0178  0.0032  0.0224 |

**Table S8.** Fukui function analysis of MO

| **Atom** | **Q(N)** | **Q(N+1)** | **Q(N-1)** | **f^-^** | **f^+^** | **f^0^** | **CCD** |
| --- | --- | --- | --- | --- | --- | --- | --- |
| 1(C)  2(C)  3(C)  4(C)  5(C)  6(C)  7(H)  8(H)  9(H)  10(H)  11(N)  12(N)  13(C)  14(C)  15(C)  16(C)  17(H)  18(C)  19(H)  20(C)  21(H)  22(H)  23(N)  24(C)  25(H)  26(H)  27(H)  28(C)  29(H)  30(H)  31(H)  32(S)  33(O)  34(O)  35(O)  36(Na) | -0.0364  -0.0417  0.0279  -0.0353  -0.0293  -0.0189  0.0447  0.0404  0.0473  0.0547  -0.0819  -0.0684  0.005  -0.0359  -0.0365  -0.0726  0.046  -0.0661  0.0429  0.0526  0.0367  0.0375  -0.046  -0.0254  0.0351  0.043  0.0353  -0.0255  0.0349  0.043  0.0347  0.4767  -0.3433  -0.3859  -0.3967  0.6072 | -0.0446  -0.0599  0.0154  -0.055  -0.0456  -0.0347  0.0391  0.03  0.0337  0.0431  -0.1244  -0.1212  0.002  -0.0548  -0.057  -0.0864  0.0359  -0.0809  0.0329  0.0268  0.0258  0.0262  -0.062  -0.0317  0.0274  0.0333  0.0273  -0.0319  0.0269  0.0333  0.027  0.4682  -0.367  -0.3897  -0.3993  0.0918 | -0.0145  -0.0134  0.047  -0.005  -0.007  0.0222  0.0606  0.0533  0.0629  0.0712  -0.0356  -0.0547  0.0667  -0.0038  -0.0074  -0.0234  0.0697  -0.0226  0.0631  0.0829  0.0612  0.0605  0.0383  -0.0055  0.0617  0.065  0.0619  -0.0057  0.0616  0.065  0.0614  0.487  -0.3146  -0.3681  -0.3702  0.6283 | 0.0218  0.0283  0.0191  0.0303  0.0222  0.0411  0.0159  0.0129  0.0156  0.0165  0.0463  0.0137  0.0617  0.0321  0.0291  0.0492  0.0238  0.0434  0.0201  0.0303  0.0245  0.023  0.0843  0.0199  0.0266  0.022  0.0266  0.0199  0.0267  0.022  0.0267  0.0103  0.0286  0.0178  0.0265  0.0211 | 0.0082  0.0182  0.0125  0.0197  0.0163  0.0159  0.0056  0.0104  0.0136  0.0116  0.0425  0.0529  0.003  0.019  0.0205  0.0137  0.0101  0.0148  0.01  0.0258  0.011  0.0113  0.016  0.0063  0.0077  0.0098  0.008  0.0064  0.008  0.0097  0.0077  0.0085  0.0238  0.0037  0.0026  0.5154 | 0.015  0.0232  0.0158  0.025  0.0193  0.0285  0.0107  0.0117  0.0146  0.0141  0.0444  0.0333  0.0324  0.0255  0.0248  0.0315  0.0169  0.0291  0.0151  0.0281  0.0177  0.0171  0.0501  0.0131  0.0172  0.0159  0.0173  0.0131  0.0174  0.0158  0.0172  0.0094  0.0262  0.0108  0.0145  0.2682 | -0.0136  -0.0101  -0.0067  -0.0106  -0.0059  -0.0252  -0.0104  -0.0026  -0.002  -0.0049  -0.0038  0.0391  -0.0587  -0.0131  -0.0086  -0.0355  -0.0137  -0.0286  -0.0101  -0.0046  -0.0135  -0.0117  -0.0683  -0.0135  -0.0189  -0.0122  -0.0187  -0.0135  -0.0187  -0.0124  -0.019  -0.0018  -0.0049  -0.0141  -0.0239  0.4943 |

**Table S9.** Economic comparison analysis of different catalysts and antibiotics

| **Sample** | **Price ($/g)** | | |
| --- | --- | --- | --- |
| Vancomycin | | 51.64 |  |
| BiOCl-250 | | 21.46 |  |

**References:**

1.	Wu, F.; Tang, Y.; Pan, Y.; Han, J.; Xing, W.; Zhang, J.; Wu, G.; Huang, Y., Interfacial Linkage Engineering Inducted Directional Electron Transfer Over ZnIn2S4@BiOCl S‐Scheme Heterojunctions for CO2 Photoreduction and Tetracycline Decomposition. *Small* **2025,** *21* (31).

2.	Chen, C.; Jiang, T.; Hou, J.; Zhang, T.; Zhang, G.; Zhang, Y.; Wang, X., Oxygen vacancies induced narrow band gap of BiOCl for efficient visible-light catalytic performance from double radicals. *Journal of Materials Science & Technology* **2022,** *114*, 240-248.

3.	Hou, J.; Dai, D.; Wei, R.; Wu, X.; Wang, X.; Tahir, M.; Zou, J.-J., Narrowing the Band Gap of BiOCl for the Hydroxyl Radical Generation of Photocatalysis under Visible Light. *ACS Sustainable Chemistry & Engineering* **2019,** *7* (19), 16569-16576.

4.	Yang, H.; Xie, J. B.; Li, X. Y.; Liu, Z.; Teng, G. X.; Zhao, Z. L.; He, J.; Zhang, C., Flower-like ZnFeO/BiOI type p-n heterojunction nanorods facilitating the efficient degradation of tetracycline. *Applied Surface Science* **2026,** *719*.

5.	Bugdayci, M.; Aksoy, S.; Kanmaz, N.; Demircivi, P., Synthesis and Characterization of Co3O4-MnO Hybrid Using Mechanical Alloying for Efficient Tetracycline Photocatalytic Degradation. *J Sustain Metall* **2026**.

6.	Zhu, W. H.; Chen, J.; Xiao, T. Q.; Qiu, Q. Q.; Bi, L. L.; Yang, K.; Liang, T. X., Novel Sm-doped FeTiO3/WO3 Z-scheme heterojunction for efficient photo-Fenton degradation of tetracycline hydrochloride. *Environmental Research* **2026,** *290*.

7.	Yang, S. Y.; Zhou, P.; Dong, M. M.; Tu, S. H., Double Type-Z Heterojunction UiO-66/ZIF-8/AgI for Highly Efficient Photocatalytic Degradation of Tetracycline. *Chemistryselect* **2026,** *11* (1).

8.	Jhelai, S.; Siva, V.; Murugan, A.; Shameem, A. S.; Pannerselvam, M.; Arunpandian, M.; Oh, T. H., Efficient visible-light photocatalytic degradation of tetracycline and methylene blue by hybrid BiFeO3@ZIF-67 nanocomposites. *Journal of Alloys and Compounds* **2026,** *1050*.

9.	Li, Y. C.; Zhang, E. R.; Tang, L. F.; Fu, Y.; Yin, S. M., Co-doped Bi2Fe4O9 microcrystals as potent peroxymonosulfate activator for efficient photocatalytic degradation of RhB. *Materials Research Bulletin* **2026,** *193*.

10.	Zhong, Y.; Ma, S.; Chen, D.; Feng, Y.; Zhang, W.; Sun, S.; Lv, G.; Zhang, W.; Zhang, J. Z.; Ding, H., Ultrathin BiOCl-OV/CoAl-LDH S-scheme heterojunction for efficient photocatalytic peroxymonosulfate activation to boost Co (IV)=O generation. *Water Res* **2024,** *258*.

11.	Hou, J.; Dai, D.; Wei, R.; Wu, X.; Wang, X.; Tahir, M.; Zou, J.-J., Narrowing the Band Gap of BiOCl for the Hydroxyl Radical Generation of Photocatalysis under Visible Light. *ACS Sustainable Chemistry & Engineering* **2019,** *7* (19), 16569-16576.

12.	Gao, M. C.; Zhang, D. F.; Pu, X. P.; Li, H.; Li, W. Z.; Shao, X.; Lv, D. D.; Zhang, B. B.; Dou, J. M., Combustion synthesis of Fe-doped BiOCl with high visible-light photocatalytic activities. *Separation and Purification Technology* **2016,** *162*, 114-119.

13.	Nayak, S.; Swain, G.; Parida, K., Enhanced Photocatalytic Activities of RhB Degradation and H2 Evolution from in Situ Formation of the Electrostatic Heterostructure MoS2/NiFe LDH Nanocomposite through the Z-Scheme Mechanism via p-n Heterojunctions. *ACS Appl Mater Interfaces* **2019,** *11* (23), 20923-20942.

14.	Hou, J.; Zhang, T.; Jiang, T.; Wu, X.; Zhang, Y.; Tahir, M.; Hussain, A.; Luo, M.; Zou, J.; Wang, X., Fast preparation of oxygen vacancy-rich 2D/2D bismuth oxyhalides-reduced graphene oxide composite with improved visible-light photocatalytic properties by solvent-free grinding. *Journal of Cleaner Production* **2021,** *328*.

15.	Kadam, A. N.; Bathula, C.; Lee, S. W., In situ growth of 1D/2D CdS-Bi2MoO6 core shell heterostructures for synergistic enhancement of photocatalytic performance under visible light. *Chemosphere* **2021,** *275*, 130086.

16.	Jamshaid, M.; Khan, H. M.; Nazir, M. A.; Wattoo, M. A.; Shahzad, K.; Malik, M.; Rehman, A. U., A novel bentonite-cobalt doped bismuth ferrite nanoparticles with boosted visible light induced photodegradation of methyl orange: synthesis, characterization and analysis of physiochemical changes. *Int J Environ an Ch* **2024,** *104* (5), 1186-1201.

17.	Zhang, Y.; Xiang, J.; Dong, H. Z.; Xie, D.; Yuan, X. S.; Deng, C. H.; Wang, F. K., Preparation of Ag-TP hybrid materials derived from MOFs and their application in photodegradation of Methyl Orange. *Materials Letters* **2025,** *397*, 138832.

18.	Abdo, S. M.; El-Hout, S. I.; Rashed, M. N.; El-Dosoqy, T. I.; El-Sheikh, S. M., Boosting visible-light photodegradation of methyl orange and ibuprofen over rGO-supported Ag3PO4 nanocomposite. *Inorg Chem Commun* **2024,** *161*, 112035.

19.	Singh, S.; Verma, N.; Umar, A.; Kansal, S. K., ZnCdS nanoparticles decorated three-dimensional MoO 3 polygonal structure: A novel photocatalyst for enhanced solar light-driven degradation of methyl orange dye. *Journal of Alloys and Compounds* **2024,** *997*, 174714.

20.	Bi, C. Y.; Han, G. Y.; Wang, Z. Y.; Yu, H. T.; Yuan, L.; Xie, Y., Rational design of type-II Co3O4/MoS2 heterojunction for enhanced visible-light-driven photocatalytic degradation of methyl orange and tetracycline hydrochloride. *Journal of Alloys and Compounds* **2025,** *1041*, 183870.

21.	Sun, H.; Lin, H.; Jia, X.; Li, X.; Li, S.; Jin, X.; Wang, Q.; Chen, S.; Cao, J., Dual structure cobalt sites on surface hydroxyl and oxygen vacancy of BiOCl for cooperative CO2 reduction and tetracycline oxidation. *Applied Catalysis B: Environment and Energy* **2024,** *359*.

22.	Lian, W.; Zhang, P.; Che, H.; Liu, B.; Ao, Y., Efficiently Piezo‐catalytic Generation of Reactive Oxygen Species on Phosphorus‐Doped BiOCl Enhancing Micropollutants Degradation. *Small* **2025,** *21* (33).

23.	Fei, C.; Hui, B.; Liu, A.; Zhou, H. Q.; Xu, T.; Chen, C. Y.; Ju, D. C.; Zhu, Z. Q.; Ma, H.; Mao, R., Study on the modification of 3D BiOCl via porous biochar and photocatalytic degradation mechanism. *Mater Today Commun* **2025,** *43*.

24.	Guo, L. H.; Li, J.; Han, S. T.; Shen, J. N.; Dai, W. X.; Wang, X. X.; Zhang, Z. Z.; Xi, H. L., The Synergy of BiOCl (110) Surface and Oxygen Vacancies for 1O2-driven Photocatalytic Durable Toluene Degradation. *Applied Surface Science* **2026,** *731*.

25.	Huang, X.; Sun, X.; Wang, F.; Xiao, J. J.; Sui, X. H.; Xu, J. Y.; Li, Y. Q.; Ye, W. N., Natural sunlight-derived RhB-sensitized BiOCl photocatalyst prepared at low temperature for large-scale wastewater treatment. *Journal of Alloys and Compounds* **2026,** *1055*.
